# Supplementary material for: Type 1 diabetes mellitus and educational attainment in childhood: a systematic review
Source: BMJ Open. 2020 Jan 26;10(1):e033215. doi: 10.1136/bmjopen-2019-033215 (PMC7045136; doi:10.1136/bmjopen-2019-033215)
Supplement: Supplementary data [file bmjopen-2019-033215supp001.pdf]

Appendix 1: Search Strategies

Search Strategy for Ovid MEDLINE (R) (1946 to present), Ovid MEDLINE(R) Epub Ahead of Print, In-Process & Other Non-Indexed Citations, Ovid EMBASE (1947 to present).

	Searches
1	exp Child/
2	exp Pediatrics/
3	exp Adolescent/
4	teen*.ti,ab.
5	child*.ti,ab.
6	adolescen*.ti,ab.
7	p?ediatric*.ti,ab.
8	juvenile*.ti,ab.
9	youth*.ti,ab.
10	(young adj3 (person* or people)).ti,ab.
11	minors.ti,ab.
12	or/1-11
13	exp Diabetes Mellitus, Type 1/
14	(type 1 diabetes or T1D or T1DM or diabet*).ti,ab.
15	(Insulin adj3 dependent).ti,ab.
16	13 or 14 or 15
17	(academic* adj3 (attain* or grade* or performance* or success* or status* or outcome* or result* or mark* or achiev* or score* or progress*)).ti,ab.
18	(educat* adj3 (attain* or grade* or performance* or success* or outcome* or result* or status* or mark* or achiev* or score* or progress*)).ti,ab.
19	(school* adj3 (attain* or grade* or performance* or success* or status* or outcome* or result* or mark* or achiev* or score* or progress*)).ti,ab.
20	exp Educational Status/
21	or/17-20
22	12 and 16 and 21
23	limit 22 to yr="2004 -Current"

Search Strategy for Thomson Reuters Web of Science

	Searches
#4	#3 AND #2 AND #1<br>(Timespan=2004-present)
#3	TS=(type 1 diabetes or t1d or t1dm or diabet* or insulin near/3 dependent)
#2	TS=(((academic* or educat* or school*) near/3 (attain* or grade* or performance* or success* or status* or outcome* or result* or mark* or achiev* or score* or progress*)))
#1	TS=(child* or p?ediatric or juvenile* or adolescen* or teen* or minors or juvenile or youth* or (young near/3 (people or person*)))

Search Strategy for EBSCO British Education Index (BEI), EBSCO Education Resources Information Center (ERIC) & EBSCO Cumulative Index to Nursing and Allied Health Literature (CINAHL)

	Searches
S10	S3 AND S6 AND S9<br>(Limiter=2004-present)
S9	S7 OR S8
S8	TI ((academic* or educat* or school*) N3 (attain* or grade* or performance* or success* or status* or outcome* or result* or mark* or achiev* or score* or progress*))
S7	AB ((academic* or educat* or school*) N3 (attain* or grade* or performance* or success* or status* or outcome* or result* or mark* or achiev* or score* or progress*))
S6	S4 OR S5
S5	AB child* or p?ediatric or adolescen* or teen* or minors or juvenile* or youth* or (young N3 (people or person*)
S4	TI child* or p?ediatric or adolescen* or teen* or minors or juvenile* or youth* or (young N3 (people or person*)
S3	S1 OR S2
S2	AB(diabet* or type 1 diabetes or T1D or T1DM)
S1	TI(diabet* or type 1 diabetes or T1D or T1DM)
