# Supplementary material for: Type 1 diabetes mellitus and educational attainment in childhood: a systematic review
Source: BMJ Open. 2020 Jan 26;10(1):e033215. doi: 10.1136/bmjopen-2019-033215 (PMC7045136; doi:10.1136/bmjopen-2019-033215)
Supplement: Supplementary data [file bmjopen-2019-033215supp002.pdf]

**Appendix 2:**

| **Journal title** |
|---|
| New England Journal of Medicine |
| Lancet |
| JAMA |
| Lancet Internal Medicine |
| BMJ |
| Diabetes Care |
| PLOS Medicine |
| Diabetologia |
| Pediatric Diabetes |
| Endocrine Reviews |
| Nature Reviews Endocrinology |
| Trends in Endocrinology & Metabolism |
| Diabetes |
| Journal of Clinical Endocrinology & Metabolism |
| Diabetes, Obesity & Metabolism |
| American Journal of Physiology – Endocrinology and Metabolism |
| Reviews in Endocrine & Metabolic Disorders |
| Journal of Endocrinology |
| European Journal of Endocrinology |
| Diabetes/ Metabolism Research and Reviews |
| Best Practice and Research in Clinical Endocrinology and Metabolism |
| Diabetes and Metabolism Journal |

**Table 1: List of key journals for which electronic table of contents were searched for additional relevant articles.**
