# Supplementary material for: Type 1 diabetes mellitus and educational attainment in childhood: a systematic review
Source: BMJ Open. 2020 Jan 26;10(1):e033215. doi: 10.1136/bmjopen-2019-033215 (PMC7045136; doi:10.1136/bmjopen-2019-033215)
Supplement: Supplementary data [file bmjopen-2019-033215supp003.pdf]

Appendix 3:

**Excluded Studies**
Abusrewil S. (2013) Epidemiology of childhood diabetes in western part of Libya (1989-2013). Pediatric Diabetes 18:117.
Almqvist C et al. (2016) Sibship and risk of asthma in a total population: A disease comparative approach. Journal of Allergy and Clinical Immunology 138(4):1219-1222e3.
Bass L (2011) Social Focus on Health and Children's Well-being. Sociological Inquiry 81: 495-498
Bezerra A et al. (2012) School attainment in childhood is an independent risk factor of dementia in late life: results from a Brazilian sample. International Psychogeriatrics 24(1): 55-61.
Bjerkedal T et al. (2006) A follow up of persons who received basic and/or supplemental benefits in childhood. Tidsskr Nor Legeforen 126: 436-439
Bortes C et al. (2018) Health problems during childhood and school achievement: Exploring associations between hospitalization exposures, gender, timing, and compulsory school grades. PLoS ONE 13(12):e0208116.
Catalano D et al. (2004) Journal of Allergy and Clinical Immunology. 155(5): 175-8.
Champaloux S, Young D (2015) Childhood Chronic Health Conditions and Educational Attainment: A Social Ecological Approach. Journal of Adolescent Health 56: 98-105
Chaudhry T, Klonoff D (2013) SMBG out of control: the need for educating patients about control solution. Diabetes Educator 39: 689-695
Compas B, et al. (2017) Neurocognitive Deficits in Children With Chronic Health Conditions. American Psychologist 72: 326-338
Cooper MN, McNamara KA, de Klerk NH, Davis EA, Jones TW (2016) School performance in children with type 1 diabetes: A contemporary population-based study. Pediatric Diabetes 17: 101-111.
Crump C, Rivera D, London R, Landau M, Erlendson B, Rodriguez E (2013) Chronic health conditions and school performance among children and youth. Annals of Epidemiology 23: 179-184.
Elrayah H, et al. (2005) Economic burden on families of childhood type 1 diabetes in urban Sudan. Diabetes Research and Clinical Practice 70: 159-165
Engelke M et al. (2008) School nurse case management for children with chronic illness: health, academic, and quality of life outcomes. Journal of School Nursing 24(4):205-214
Erkolahti R, Ilonen T (2005) Academic Achievement and the Self-Image of Adolescents with Diabetes Mellitus Type-1 And Rheumatoid Arthritis. Journal of Youth and Adolescence 34: 199.
Fraser A et al. (2012) Associations of existing diabetes, gestational diabetes, and glycosuria with offspring IQ and educational attainment: The avon longitudinal study of parents and children. Experimental Diabetes Research 963735.
Gonzalez A, et al. (2012) Childhood and family influences on depression, chronic physical conditions, and their comorbidity: Findings from the Ontario Child Health Study. Journal of Psychiatric Research 46: 1475-1482
Hassan M et al. (2017) Assessment of health-related quality of life in Egyptian adolescents with type 1 diabetes: DEMPU survey. Journal of Pediatric Endocrinology and Metabolism 30(3): 277-283.
Jameson P et al. (2006) Diabetes, cognitive function, and school performance. School Nurse News 23(3):34-36.
Jesic M et al. (2013) School problems in children and adolescents with type 1 diabetes. Pediatric Diabetes 18:102-103.
Lansing A et al. (2018) Academic achievement and metabolic control in adolescents with type 1 diabetes, Children's Health Care 47(1):16-33
Liaqat A at al. (2017) Epidemology of type-I diabetes and its economic burden on society. Pakistan Journal of Medical and Health Sciences 11(2):714-717.
Lynch P et al. (2004) Predicting academic achievement in youths with type 1 diabetes: A biopsychosocial model. Diabetes 53:A436.
Maslow G et al. (2011) Growing up with a chronic illness: social success, educational/vocational distress. Journal of Adolescent Health 49(2):206-12.
Maslow G et al. (2012) Protective connections and educational attainment among young adults with childhood-onset chronic illness. 82(8):364-70.

Meo SA, Alkahlan MA, Al-Mubarak MA, et al. (2013) Impact of type 1 diabetes mellitus on academic performance. Journal of International Medical Research 41: 855-858
Merrick H et al. (2015) Characteristics of young people with long term conditions close to transfer to adult health services. BMC health services research 15:435.
Milovanovic I et al. (2012) Entred-Ado study: Health, education and risk behaviours of adolescents with diabetes. Diabetologia 1:S140.
Mitosi N et al. (2013) Family role in treatment adherence of type 1 diabetes: Research data from Greek population. Pediatric Diabetes 18:110.
Nasuuna E, Santoro G, Kremer P, de Silva AM (2016) Examining the relationship between childhood health conditions and health service utilisation at school entry and subsequent academic performance in a large cohort of Australian children. Journal of Paediatrics and Child Health 52: 750-758
Neves C et al. (2013) Obesity, diet and physical activity: The reality of our teenagers. Atencion Primaria 2:84.
Nielson H et al. (2016) Type 1 diabetes, quality of life, occupational status and education level - A comparative population-based study. Diabetes Research & Clinical Practice 121:62-68
Ovesen L et al. (2015) Education level, occupational status and quality of life in adults with type 1 diabetes and in the general population: A comparative study. Diabetologia 1:S412.
Roman R et al. (2016) School performance in children and adolescents with type 1 diabetes. Hormone Research in Paediatrics 86:51.
Roman R et al. (2017) Metabolic control and school performance in children with type 1 diabetes. Revista Chilena de Pediatria 88(5):586-594.
Ryan C. (2012) Does severe hypoglycaemia disrupt academic achievement in children with early onset diabetes? Developmental Medicine and Child Neurology 54(5):393-394
Schiel R et al. (2016) Plasma copeptin in children and adolescents with type 1 diabetes mellitus in comparison to healthy controls. Diabetes Research and Clinical Practice 118:156-161.
Steen Carlsson K et al. (2015) Type 1 diabetes with early onset and educational field at upper secondary and university level: Is own experience an asset for a health care carrier? Diabetologia 1:S411.
Tahirovic H et al. (2013) Impact of socioeconomic status on health-related quality of life in children with type 1 diabetes mellitus in Bosnia and Herzegovina. Minerva Pediatrica 65(2):207-212.
Taras H et al. (2005) Chronic health conditions and student performance at school. Journal of School Health 75(7):255-266.
von Stumm S et al. (2011) Childhood behavior problems and health at midlife: 35-year follow-up of a Scottish birth cohort. Journal of child psychology and psychiatry and allied disciplines 52(9):992-1001.
Wennick A et al. (2011) Attained education and self-assessed health later in life when diagnosed with diabetes in childhood: a population-based study. Pediatric Diabetes 12(7):619-26.
Winnick J et al. (2017) Metabolic Control and Academic Achievement Over Time Among Adolescents With Type 1 Diabetes. School Psychology Quarterly 32(1):105-117.

Table 1: List of excluded studies following full text review.
