# Supplementary material for: Type 1 diabetes mellitus and educational attainment in childhood: a systematic review
Source: BMJ Open. 2020 Jan 26;10(1):e033215. doi: 10.1136/bmjopen-2019-033215 (PMC7045136; doi:10.1136/bmjopen-2019-033215)
Supplement: Supplementary data [file bmjopen-2019-033215supp004.pdf]

Appendix 4:

	**Newcastle Ottawa Scale**				**Overall Risk of Bias**
**Study**	**Selection**	**Comparability**	**Outcome**	**Overall**	
**Dahlquist & Kallen (2007)**	4/4	2/2	2/3	**8/9**	**Low**
**Persson et al. (2013)**	4/4	2/2	3/3	**9/9**	**Low**

Table 1: Newcastle Ottawa Scale ratings for included studies.
