# Supplementary material for: Type 1 diabetes mellitus and educational attainment in childhood: a systematic review
Source: BMJ Open. 2020 Jan 26;10(1):e033215. doi: 10.1136/bmjopen-2019-033215 (PMC7045136; doi:10.1136/bmjopen-2019-033215)
Supplement: Author's manuscript [file bmjopen-2019-033215.draft_revisions.pdf]

BMJ Open is committed to open peer review. As part of this commitment we make the peer review history of every article we publish publicly available.

When an article is published we post the peer reviewers' comments and the authors' responses online. We also post the versions of the paper that were used during peer review. These are the versions that the peer review comments apply to.

The versions of the paper that follow are the versions that were submitted during the peer review process. They are not the versions of record or the final published versions. They should not be cited or distributed as the published version of this manuscript.

BMJ Open is an open access journal and the full, final, typeset and author-corrected version of record of the manuscript is available on our site with no access controls, subscription charges or pay-per-view fees (http://bmjopen.bmj.com).

If you have any questions on BMJ Open's open peer review process please email info.bmjopen@bmj.com

# Type 1 Diabetes Mellitus and Educational Attainment in Childhood: A Systematic Review



| | |
|---|---|
| Journal: | *BMJ Open* |
| Manuscript ID | bmjopen-2019-033215 |
| Article Type: | Research |
| Date Submitted by the Author: | 25-Jul-2019 |
| Complete List of Authors: | Oakley, Natalie; Cardiff University School of Medicine, Division of Population Medicine<br>Kneale, Dylan; UCL Institute of Education,<br>Mann, Mala; Cardiff University, University Library Services<br>Hilliar, Mariann; University Hospital of Wales, Cardiff University Library<br>Dayan, Colin; Cardiff University School of Medicine<br>Gregory, John; Cardiff University School of Medicine, Division of Population Medicine<br>French, Robert; Cardiff University School of Medicine |
| Keywords: | DIABETES & ENDOCRINOLOGY, Paediatric endocrinology < DIABETES & ENDOCRINOLOGY, Community child health < PAEDIATRICS |
| | |



SCHOLARONE™
Manuscripts

# Type 1 Diabetes Mellitus and Educational Attainment in Childhood: A Systematic Review

Oakley N[1], Kneale D[2], Mann M[3], Hilliar M[4], Dayan C[5], Gregory JW[1], French R*[5]

*Corresponding author: Dr Robert French. Email: frenchr3@cardiff.ac.uk

[1]Division of Population Medicine, Cardiff University School of Medicine, Neuadd Meirionnydd, University Hospital Wales, Cardiff, CF14 4YS.

[2] Social Science Research Unit (SSRU), University College London, 18 Woburn Street, London, WC1H 0NR

[3]Specialist Unit for Review Evidence (SURE), Cardiff University School of Medicine, Neuadd Meirionnydd, University Hospital Wales, Cardiff, CF14 4YS.

[4] Cardiff University Library, Cochrane Building, University Hospital Wales, Heath Park, CF14 4YU.

[5] Cardiff University School of Medicine, University Hospital Wales, Heath Park, Cardiff, CF14 4YS.

Word count: 3407

For peer review only



For peer review only - http://bmjopen.bmj.com/site/about/guidelines.xhtml

# ABSTRACT

**Objectives**

Type 1 diabetes mellitus (T1DM) has the potential to affect children's educational attainment through clinical and social pathways. The primary objective of this systematic review was to evaluate available literature on whether T1DM has an impact on educational attainment in individuals undertaking high stakes standardised testing at the end of compulsory schooling.

**Methods**

We conducted a systematic review of studies comparing educational attainment for individuals with and without a diagnosis of T1DM who have undertaken high stakes testing at the end of compulsory schooling. Seven databases were searched to identify peer-reviewed and grey literature available from January 2004. Two reviewers performed study selection and data extraction independently. Quality and risk of bias in the observational studies included were assessed using the Newcastle-Ottawa Scale. We undertook a detailed narrative synthesis of the included studies.

**Results**

3,103 articles were identified from the database search, with two papers (using the same linked administrative data) meeting final inclusion criteria. Both papers were Swedish cohort studies found to be of high quality with low risk of bias. A small but statistically significant difference was reported in mean final grades at the end of compulsory schooling, with children with T1DM found to have lower mean grades than their non-diabetic counterparts.

**Conclusions**

More contemporary research is required to evaluate the impact of T1DM in childhood on educational attainment in individuals undertaking high stakes standardised testing at the end of compulsory schooling, taking into consideration the substantial advances in treatment and management of T1DM in the last decade.



**Keywords**

Diabetes mellitus type 1; Educational status; Achievement; Adolescent; Child; School; Systematic Review

**PROSPERO Registration number**

CRD42017084078




For peer review only - http://bmjopen.bmj.com/site/about/guidelines.xhtml

# STRENGTHS AND LIMITATIONS OF THIS STUDY

- Previous studies have found an association between T1DM in childhood and reduced cognitive function, however there is less evidence assessing the effect of T1DM on educational attainment, particularly in the form of high stakes examinations.
- This review has comprehensively evaluated available literature reporting the impact of T1DM on educational attainment in individuals undertaking high stakes standardised testing at the end of compulsory schooling.
- Our findings were reported using the Cochrane Handbook for Systematic Reviews of Interventions and recommended methods and checklist of the Preferred Reporting Items for Systematic Reviews and Meta-Analyses (PRISMA), with the quality of evidence assessed using the Grading of Recommendations Assessment, Development and Evaluation (GRADE) system.
- Study selection and data extraction were performed independently by two reviewers, and the quality and risk of bias of the studies included in this review were assessed using the Newcastle-Ottawa Scale (NOS).
- Limitations of this review include the use of narrow inclusion thresholds, commensurate with our research objectives, resulting in a small number of included studies thereby precluding the use of meta-analysis and limiting generalisability of the results.

For peer review only



For peer review only - http://bmjopen.bmj.com/site/about/guidelines.xhtml

# INTRODUCTION

**Background & Rationale**

Type 1 diabetes mellitus (T1DM) is an autoimmune disorder which destroys the insulin-producing beta cells in the pancreas, preventing the body from adequately regulating blood glucose levels.(1) According to the 2017 IDF Diabetes Atlas 8th edition, the number of children diagnosed with type 1 diabetes is increasing annually, with an estimated 96,100 new cases worldwide every year.(2)

Physiological complications of diabetes can include hypoglycaemia, hyperglycaemia & ketoacidosis.(1) These complications, especially if recurrent, have the potential to impact on educational attainment via a combination of mechanisms including altered cognitive function and non-attendance for acute treatment.(3, 4) Glucose is the principal energy source of the human brain. Hypoglycaemia, particularly if severe or recurrent, can result in neuroglycopenia which, in turn, may result in neuronal injury and subsequent cognitive impairment.(5) Hyperglycaemia, particularly when associated with ketoacidosis, has also been found to have detrimental effects on the brain via damage to white matter, disrupted functioning of the blood-brain barrier and transient focal cerebral oedema. These changes can again impair cerebral functioning resulting in cognitive impairment.(6) There is also the potential for attendance and attainment to be affected by hospital appointments or illness at critical points in the educational trajectory.(7) This is especially important when considering high stakes testing at the end of compulsory schooling which has the potential to significantly impact future social and economic outcomes, such as social status, employment and income(8).

Many patients and their families report worries about the support available for children with diabetes at school and concerns about the potential negative impact that T1DM may have on school attendance.(4, 7) In an observational study published in 2007, Amillategui et al. utilised self-reporting questionnaires to identify specific parental concerns regarding T1DM in the school setting. Many of these concerns focused on aspects such as glycaemic control, social integration, medication administration and understanding of staff members, which may affect children's attainment, wellbeing and experience in school.(9)

Previous literature has studied the effects of T1DM on cognitive functioning in children. In a review published in 2004, Desrocher et al. found an association between T1DM in childhood and poorer neurocognitive outcomes, with the most significant deficits found to be related to age of disease onset, episodes of hypoglycaemia and hyperglycaemia around the time of puberty.(10) In 2008, Gaudieri et al reported that T1DM in childhood was associated with poorer performance in learning and reduced memory skills.(11) Further studies published by Naguib et al. in 2008(12) and He et al. in 2018(13) reported similar findings, suggesting a detrimental impact of T1DM in childhood on cognitive function. However, there is less evidence as to the effect T1DM has on educational attainment, particularly in the form of high stakes examinations, and the magnitude of this impact.(7)

Each of the four home nations within the United Kingdom has specific legislation focused on support for children and young people with additional learning needs and medical conditions including T1DM in school.(14-19) Quantifying the impact of T1DM on educational attainment at the end of compulsory



For peer review only - http://bmjopen.bmj.com/site/about/guidelines.xhtml

schooling and identifying domains most affected may be useful in assessing what and how much support should be focused on children with T1DM in school.

The primary objective of this review was to identify and analyse the available literature on whether T1DM has an impact on educational attainment in individuals undertaking high stakes standardised testing at the end of compulsory schooling.

Secondary objectives included assessment of the effect of T1DM on school attendance and educational attainment at other stages on the educational trajectory if reported.

## METHODS

The protocol for this systematic review is registered with PROSPERO (International Prospective Register of Systematic Reviews) at the NHS Centre for Reviews and Dissemination (CRD) at the University of York (Registration number CRD42017084078)(20) and has been previously published.(21) The Cochrane Handbook for Systematic Reviews of Interventions(22) and recommended methods and checklist of the Preferred Reporting Items for Systematic Reviews and Meta-Analyses (PRISMA)(23) were used to structure this systematic review. Patients were not involved in the development and completion of this systematic review.

Studies were included if they fulfilled the following inclusion criteria: 1) observational study (including prospective and retrospective cohort and case-control studies) or randomised controlled trial; 2) included individuals who have undertaken high stakes testing at the end of compulsory schooling; and 3) compared the grades obtained by individuals with type 1 diabetes with a representative population control. The primary outcome assessed was grade obtained following high stakes testing at the end of compulsory schooling. Secondary outcomes were school attendance and grades obtained at other stages on the educational trajectory.

Comprehensive electronic literature search strategies were developed to search the following seven databases: Ovid MEDLINE (1946 to present), Ovid MEDLINE (Epub Ahead of Print, In-Process & Other Non-Indexed Citations), Ovid EMBASE (1947 to present), Thomson Reuters Web of Science, EBSCO British Education Index (BEI), EBSCO Education Resources Information Center (ERIC) and EBSCO Cumulative Index to Nursing and Allied Health Literature (CINAHL). Only studies published since January 2004 in the English language were considered. The initial search was undertaken on 15$^{th}$ January 2018 and then updated on 17$^{th}$ January 2019 to identify any further studies. The results for the initial and updated searches were combined. The search strategies used for the above databases are given in Appendix 1.

To identify (i) studies in progress, (ii) unpublished research, or (iii) research reported in the grey literature, we searched electronic Table of Contents (eTOC) of key journals (listed in Appendix 2) and trial registries (24-26) for studies published within the last two years. Finally, we searched for studies via relevant websites including Diabetes.org.uk and jdrf.org.uk, review articles, reference lists and citation tracking of included studies.



For peer review only - http://bmjopen.bmj.com/site/about/guidelines.xhtml

For peer review only

After removal of duplicates, two independent reviewers (NO & RF) screened study titles and abstracts using Eppi-Reviewer 4.0.(27) Full texts of potentially eligible records were then screened according to set inclusion criteria. The rationale for the exclusion of studies at each stage was documented. The remaining included studies underwent data extraction by NO & RF independently.

The quality and risk of bias in the observational studies included in this review were assessed by NO & RF independently using the Newcastle-Ottawa Scale (NOS).

The principle continuous outcome measure used in included studies was the 'mean difference' between school grades attained. When assessing the likelihood of achieving different grades, the principle binary outcome measure used was the 'odds ratio'. Meta-analysis was not possible because of the substantial duplication of data linkage from the databases, with both papers including a cohort of participants born 1973 to 1978. As a result, a detailed narrative synthesis of the included papers was undertaken.

The quality of evidence for the primary outcome was assessed using the Grading of Recommendations Assessment, Development and Evaluation (GRADE) system.(28) Results are presented in a 'Summary of findings' table as recommended by the Cochrane Handbook for Systematic Reviews of Interventions.(22)

## RESULTS

The database search identified 3,103 papers. After duplicates were removed, 2,304 articles underwent abstract screening (Figure 1). A total of 45 papers were shortlisted for full-text review. No full text was available for nine studies, and further information was required for one study. The authors of these studies were contacted, but as no response or clarification was obtained, these studies were excluded.

Table 1 lists the reasons for exclusion for the studies rejected on full-text review (full citations listed in Appendix 3). The abstracts of four additional studies identified from the reference lists of the studies shortlisted for full-text review were screened and rejected.

For peer review only

### Table 1: Primary reasons for exclusion of studies following full-text review

| Reason for exclusion | | | | | |
|---|---|---|---|---|---|
| **No final school-based grades assessed** | **No full text available** | **Study not specifically assessing T1DM** | **Not assessing grades following high stakes tests at end of compulsory schooling** | **No comparison between children with and without T1DM** | **Study design not in scope** |
| Bass L. 2011<br>Bjerkedal T et al. 2006<br>Champaloux S et al. 2015<br>Chaudhry T et al. 2013<br>Compas B et al. 2017<br>Elrayah H et al. 2005<br>Gonzalez A et al. 2012<br>Hassan M et al. 2017<br>Liaqat A at al. 2017<br>Maslow G et al. 2011<br>Maslow G et al. 2012<br>Merrick H et al. 2015<br>Neves C et al. 2013<br>Nielson H et al. 2016<br>Ovesen L et al. 2015<br>Ryan C. 2012<br>Schiel R et al. 2016<br>von Stumm S et al. 2011<br>Wennick A et al. 2011 | Abusrewil S. 2013<br>Catalano D et al. 2004<br>Jesic M et al. 2013<br>Lynch P et al. 2004<br>Milovanovic I et al. 2012<br>Mitosi N et al. 2013<br>Roman R et al. 2016<br>Steen Carlsson K et al. 2015<br>Tahirovic H et al. 2013 | Almqvist C et al. 2016<br>Bezerra A et al. 2012 | Cooper M et al. 2016<br>Crump C et al. 2013<br>Erkolahti R et al. 2005<br>Lansing A et al. 2018<br>Meo S et al. 2013<br>Nasuuna E et al. 2016<br>Roman R et al. 2017 | Engelke M et al. 2008<br>Winnick J et al. 2011<br>Bortes C et al. 2018 | Fraser A et al. 2012<br>Jameson P et al. 2006<br>Taras H et al. 2005 |

Two papers met all of the inclusion criteria, both electronic population cohort studies from Sweden (3, 29), as detailed in Table 2 below. The most recent sought to improve on the analysis of the earlier paper by (i) using different methods, (ii) using more refined model specifications and (iii) adding later life outcomes. Both papers compared mean attainment on a five-point scale (1-5) at age 16 years for those with diabetes to those without diabetes. The sample size decreased for the later study because they used more restricted birth cohorts to allow comparisons of labour force outcomes aged 29 years.

### Table 2: Characteristics of included observational studies.

| Author (year) | Design | Country | No. cases (known T1DM) | No. controls (no T1DM) | Matching completed (Y/N) | Controlled confounders |
|---|---|---|---|---|---|---|
| Persson et al. (2013) | Electronic population cohort | Sweden | 2,392 | 9,563 | Y | Year of birth, Gender, School year, Level of parental education, Parental income, Parental country of origin |



For peer review only - http://bmjopen.bmj.com/site/about/guidelines.xhtml

| | | | | | | |
|---|---|---|---|---|---|---|
| Dahlquist and Kallen (2007) | Electronic population cohort | Sweden | 5,159 | 1,330,968 | N | Year of birth, Gender, Maternal age, parity and educational level |

The primary outcome in these two studies was mean final grade across all school subjects, from compulsory secondary school (Table 3). Persson states that these grades are teacher-rated, therefore potentially introducing bias if teachers subconsciously adjust grades because of diabetes status. For the later exam years (1998-2003) used by Dahlquist, the outcome scale was changed and estimates for this period were given separately by subject only. Both papers show a similar negative effect of diabetes on mean attainment, after adjusting for confounders. Dahlquist estimates children with T1DM achieve 0.08 fewer final mean marks, while Persson estimates children with T1DM achieve 0.07 fewer final mean marks. Persson also use quantile regression to show that the effect of diabetes is strongest in the lowest quantile of attainment. Both studies also break down the mean attainment score to consider marks in four core subjects- Maths, Swedish, English and Sports.

**Table 3: Effect of T1DM on mean final grade of 1) all school subjects and 2) specific subjects (Maths, Swedish, English, Sports) at the end of compulsory school.**

| | **Dahlquist and Kallen** | **Persson et al.** |
|---|---|---|
| 1) Mean final grade of all school subjects from compulsory schooling at age 16 (Mean final grade) | T1DM:<br>3.15 (adjusted)<br>No T1DM:<br>3.23 (adjusted)<br>Mean difference (adjusted): 0.08<br><br>T1DM found to have negative impact on mean grade attainment of all school subjects. | T1DM:<br>3.13 +/- 0.75 (unadjusted)<br>No T1DM:<br>3.21 +/- 0.72 (unadjusted)<br>Mean difference (adjusted): 0.07 +/- 0.02, p<0.001.<br>T1DM found to have negative impact on mean grade attainment of all school subjects. |
| Additional statistical analysis performed | Mean t value -0.24 +/- 0.04<br>t* value -5.19 p<0.001 | Quantile regression - Negative effect of T1DM greatest in the lowest quantile of attainment, diminishing with increasing attainment quantiles, becoming statistically insignificant for the highest attaining quantile. |
| 2) Mean final grade of specific subjects (Maths, Swedish, English and Sports) from compulsory school at age 16 | Odds ratios used to compare attainment of each grade in specific subjects in order to estimate effect of T1DM.<br>• Sports/Athletics: Clear negative association seen between T1DM & grade attainment.<br>• English: Similar pattern to Sports/Athletics however weaker gradient.<br>• Maths and Swedish: Children with T1DM found to be more likely to achieve lower grades however no clear differences were seen for high grades. | Mean difference in attained grades (unadjusted), predicted probability of achieving grades 1-5 in specific subjects, and odds ratios using ordered logistic regression used to estimate effect of T1DM.<br>• Sports/Athletics: Clear negative association seen between T1DM & grade attainment.<br>• Similar trend seen in all subjects - largest differences in attainment seen in Sports/Athletics, smallest differences seen in Maths. |



For peer review only - http://bmjopen.bmj.com/site/about/guidelines.xhtml

There was a clear gradient found for children with diabetes in Sports/Athletics, with children with T1DM found to be more likely to achieve lower grades and less likely to achieve higher grades. The quality of evidence for these outcomes, assessed using the Grading of Recommendations Assessment, Development and Evaluation (GRADE) system(28), is presented in the 'Summary of findings' table (Table 4).

For peer review only



For peer review only - http://bmjopen.bmj.com/site/about/guidelines.xhtml

## Table 4: Summary of Findings Table

**The Association between Type 1 Diabetes Mellitus and Educational Attainment in Childhood**

**Patient or population**: Individuals who have undertaken high stakes testing at the end of compulsory schooling, under 18 years of age.
**Setting**: School
**Intervention**: Known diagnosis of T1DM.
**Comparison**: No known diagnosis of T1DM.

| Outcomes | Impact | № of participants (studies) | The certainty of the evidence (GRADE) |
|---|---|---|---|
| Mean final grade of all school subjects from compulsory schooling at age 16 (Mean final grade) | Both papers found significantly lower attainment of between 0.07-0.08 marks achieved by children with T1DM compared with their non-diabetic counterparts. | (2 observational studies) | ⊕◯◯◯ VERY LOW [a,b,c,d] |
| Mean final grade of specific subjects (Maths, Swedish, English and Sports) from compulsory school at age 16 | **Dahlquist and Kallen** test for differences in the odds for each level of attainment by subject in the raw categories and in an alternative four categories where the raw numeric scores (1988-97) were manipulated to have the same number of categories as the alphabetical marks (1998-2003). There was a clear gradient for children with diabetes in Sports (greater odds of a poor score, lower odds of a good score), in Maths and Swedish, children with diabetes had a higher odds of a low score (no clear differences for high scores), and there were no clear effects for English.<br><br>**Persson et al** show a similar pattern, though all the comparisons of levels within subjects are statistically significant (though comparison for the mean attainment scores for Maths was not significant), with the largest differences in attainment to be for Sports and the smallest differences for Maths. | (2 observational studies) | ⊕◯◯◯ VERY LOW [a,b,c,d] |

**GRADE Working Group grades of evidence**
**High certainty:** We are very confident that the true effect lies close to that of the estimate of the effect
**Moderate certainty:** We are moderately confident in the effect estimate: The true effect is likely to be close to the estimate of the effect, but there is a possibility that it is substantially different
**Low certainty:** Our confidence in the effect estimate is limited: The true effect may be substantially different from the estimate of the effect
**Very low certainty:** We have very little confidence in the effect estimate: The true effect is likely to be substantially different from the estimate of effect

*Explanations*
a. Both included studies control for gender, academic year and maternal education. Dahlquist and Kallen additionally condition on maternal age and parity, while Persson et al additionally control for paternal education, long-term parental income and parental country of origin.
b. Heterogeneity could not be reliably assessed as only two papers met final inclusion criteria (1 study with updated methodology). As a result, there is a substantial amount of duplication of cases from the databases, with both papers including a cohort of participants born 1973 to 1978.
c. Publication bias could not be reliably assessed as only two papers met final inclusion criteria (one study with updated methodology).



For peer review only - http://bmjopen.bmj.com/site/about/guidelines.xhtml

d. Total no. of participants unable to be combined because of the unknown quantity of data duplication from the same cohort of participants born 1973 to 1978. (With T1DM – Dahlquist N=5,159 & Persson N=2,392. Without T1DM – Dahlquist N=1,330,968, Persson N=9,563).

For peer review only

Both studies estimate the effect of age of diagnosis using further models, suggesting lower grade attainment with earlier diagnosis. Dahlquist estimates conditional means for age groups of diagnosis (<2, 2-5, 5-10, 10-15 years), reporting the largest (but non-significant) differences for the earliest diagnosis group vs later diagnosis groups of between 0.11 to 0.20 marks. Persson uses those without diabetes as the reference group, showing a negative association between earlier diagnosis and attainment. Those diagnosed aged 10-15 were found to have 0.06 lower attainment, those diagnosed aged 5-9 were found to have 0.07 lower attainment, and those diagnosed aged 0-4 were found to have 0.15 lower attainment.

The quality and risk of bias for the included studies was assessed as high-quality methodology with low risk of bias using the Newcastle-Ottawa Scale (NOS). Full NOS ratings can be seen in Appendix 4. Persson scored full marks across the three domains. Dahlquist lost one mark on the outcome assessment domain for not reporting the numbers lost due to linkage (although they do consider the reasons why cases may not be included). Despite both papers being judged to be methodologically sound overall, the certainty of the evidence was deemed to be very low, mainly reflecting the small number of studies identified and available for analysis.

# DISCUSSION

**Summary of evidence**

This systematic review identified two papers (using overlapping Swedish administrative cohorts) assessing the association between type 1 diabetes mellitus in childhood and educational attainment following high stakes testing at the end of compulsory schooling. Both papers found statistically significant lower mean attainment of between 0.07-0.08 marks in those with T1DM. Persson checked this relationship by attainment quantile and showed that the differences were greater for lower attainment levels. Both papers considered within-subject attainment and found a negative effect of T1DM. This effect was particularly pronounced in Sports/Athletics but less clear in Maths. It is of great concern that the largest negative association was seen in Sports/Athletics because exercise is a key tool for managing blood sugar and suggests other potential confounding factors such as parental or teacher anxiety regarding the involvement of diabetic children in physical education at school and the risk of hypoglycaemia. Both papers suggested that the negative effect of diabetes on mean attainment was greater for earlier diagnosis, though these effects were only found to be significant in Persson. This hints that the mechanisms driving the differences are not equal for all children with diabetes. Persson also found that the differences in attainment between those with and without diabetes are greater for low ability students.

**Limitations**

**Limitations of included studies**

With only two papers based on overlapping datasets, it is difficult to generalise to other populations, particularly when the findings of the included studies relate to a single country with a particularly high prevalence rate of T1DM and excellent diabetes-related outcomes(2).

The included studies controlled for demographics and socio-economic characteristics, but there are other unobserved covariates which should be considered. Other studies considered living arrangements, country of birth of the child, main language spoken at home, ability to speak English, attendance in preschool, and



For peer review only - http://bmjopen.bmj.com/site/about/guidelines.xhtml

parents' occupation.(30) While the included studies were able to adjust for age of diagnosis, they did not have data for other clinical aspects of diabetes, particularly the quality of diabetes management as measured by HbA1c. Cooper et al.(7) were able to show that a higher HbA1c (mean HbA1c during the two years before testing) was associated with poorer attainment, while history of severe hypoglycaemia or history of diabetic ketoacidosis showed no effect on educational attainment.

Finally, neither of the included studies included our secondary outcome, school attendance. In an excluded study, Cooper et al.(7) showed significantly lower school attendance in children with type 1 diabetes in all age groups ($p < 0.001$); from 2.6% lower attendance in year three up to 3.4% lower attendance in year five. This study and that of Crump et al.(31) also used attendance as a covariate in models of attainment.

**Limitations of review**

The primary limitation of the review is that the thresholds for inclusion were very narrow, meaning that only two studies met our inclusion criteria. The small number of studies (with overlapping individuals) meeting our inclusion criteria precluded the use of meta-analysis or other evidence synthesis techniques. While the use of such narrow inclusion criteria was commensurate with our research aim, as well as being consistent with the protocol for the review,(21) future systematic reviewers may consider the way in which the following criteria limited the number of studies included.

The first limiting restriction was including only studies modelling attainment from 'high stakes testing at the end of compulsory schooling'. Although this is justified through the impact of these tests on adult life chances, this criteria excluded studies which have otherwise suitable study designs for exploring the association between diabetes and educational outcomes, such as Cooper et al.(7) This restriction also excluded studies which lack national standardised testing or administrative data records, either because they considered cohorts prior to such testing regimes(32) or were studies from countries without such testing regimes.(33, 34)

The second limiting restriction was excluding publications before 2004 in view of changes in the treatment of T1DM. The 2015 NICE guidelines(35) state that since 2004 there have been major changes in routine management of type 1 diabetes. Whilst this is pertinent to the UK and most Western countries, this date was perhaps less appropriate for developing counties. In addition, studies published since 2004 were based on cases from earlier years before those changes, for example, the cohorts for our included studies were born 1972-85. This shows that more contemporaneous studies are required to assess the impact of these treatment changes. For example, Cooper et al.(7) includes data for children born up to 2003, and so can report insulin pump therapy being associated with better school performance than twice daily injections.

The third limiting restriction was requiring representative control cases which allow estimates that are generalisable to wider populations, for example excluding studies recruiting from a single clinic. As for the restriction on the outcome measure, this excludes countries without registries or linked data. This may lead to biases when the countries with high quality linked data, such as the Nordic countries, are also those with high diabetes prevalence.

One related consideration is the use of matching or population controls. In the two included studies these methods are used without justification of the choice or the matching specifications such as the number of controls per case. Matching is traditionally done to make the treatment and control groups more similar. However, these papers do not provide any evidence or reason why those children with type 1 diabetes would



For peer review only - http://bmjopen.bmj.com/site/about/guidelines.xhtml

For peer review only

be substantively different in terms of other factors which may influence educational attainment. One motivation for the matching approach is to give the appearance of having well-matched healthy controls (regardless of the sparsity of variables used in the process). However, other diagnosed (or undiagnosed) comorbidities within this cohort may affect educational attainment and should be taken into consideration. For example Meo et al.(34) exclude cases with a range of health conditions (reported tobacco use, gross anaemia, tuberculosis, rheumatic fever, rheumatoid arthritis, vision problems, hearing problems or behavioural problems, use of any medication or hospital admissions other than for diabetes mellitus) to reduce the effect of other co-morbidities within the cohort. An alternative to matching with healthy controls is to compare attainment for children with T1DM with children with other health conditions, in order to evaluate the mechanisms through which diabetes affects attainment. For example, asthma may affect sleep and concentration, but have less of an impact on attendance, while epilepsy may share some elements of the social stigma, but have a lower effect on functioning at school.

# CONCLUSION

The papers identified in this paper show a weak negative association between diabetes and overall attainment, with differences in the gradient for specific subjects (greatest in Sports/Athletics and smallest for Maths) and attainment groups (greatest for lowest ability). The trend of increasing prevalence of T1DM makes the need for more robust evidence more pressing. More studies from a broader range of contexts are required, including studies utilising alternative methods to provide evidence from countries where large-scale data linkage is not a viable approach. In addition, studies using more contemporaneous data, acknowledging the substantial advances in management of T1DM in the last decade, and ideally linking clinical data such as HbA1c with educational outcomes, are required to aid future recommendations relating to support at school for children with T1DM, especially in the setting of high stakes testing at the end of compulsory schooling.

For peer review only



For peer review only - http://bmjopen.bmj.com/site/about/guidelines.xhtml

# FUNDING

This research was supported in part by the MRC grant MR/N015428/1.


# DUALITY OF INTEREST

All authors have completed the ICMJE uniform disclosure form at www.icmje.org/coi_disclosure.pdf and declare: RF has received a grant from the Medical Research Council MR/N015428/1 for his work as principal investigator of the project 'Investigating the inter-relationship between diabetes and children's educational achievement'. All authors have no conflict of interest to report; no financial relationships with any organisations that might have an interest in the submitted work in the previous three years; no other relationships or activities that could appear to have influenced the submitted work.

# CONTRIBUTION STATEMENT

RF is the review guarantor. The concept of the review was proposed by RF and JG; the manuscript was drafted by NO and RF, and edited by all authors. MH, NO and RF designed the search strategy with advice from MM. NO, MM and RF contributed to the development of the study eligibility criteria and data extraction criteria. JG and CD provided expertise on type 1 diabetes. MM provided expertise on systematic review methodology. NO and RF screened the titles and abstracts of records retrieved from the searches using the predetermined inclusion criteria, with any doubt or disagreement discussed with JG. NO and RF independently completed data extraction and assessed the quality and risk of bias of the included studies using the Newcastle-Ottawa Scale (NOS), consulting JG if any disagreements. DK provided expertise on data extraction & analysis. All authors read, edited and approved the final manuscript.

For peer review only



For peer review only - http://bmjopen.bmj.com/site/about/guidelines.xhtml

## REFERENCES

1. Holt R, Cockram C, Flyvbjerg A, Goldstein B. Textbook of Diabetes, 5th edition. West Sussex, UK: John Wiley and Sons Ltd.; 2017.
2. International Diabetes Federation. IDF Diabetes Atlas, 8th edition. Brussels, Belgium: International Diabetes Federation; 2017. Available from: http://www.diabetesatlas.org. (Accessed 30th August 2018)
3. Persson S, Dahlquist G, Gerdtham UG, Steen Carlsson K. Impact of childhood-onset type 1 diabetes on schooling: a population-based register study. *Diabetologia* 2013;56(6):1254-62.
4. Streisand R, Monaghan M. Young Children with Type 1 Diabetes: Challenges, Research, and Future Directions. *Current diabetes reports* 2014;14(9):520.
5. Graveling AJ, Deary IJ, Frier BM. Acute Hypoglycemia Impairs Executive Cognitive Function in Adults With and Without Type 1 Diabetes. *Diabetes Care* 2013;36(10):3240-6.
6. Cameron FJ, Scratch SE, Nadebaum C, Northam EA, Koves I, Jennings J, et al. Neurological Consequences of Diabetic Ketoacidosis at Initial Presentation of Type 1 Diabetes in a Prospective Cohort Study of Children. *Diabetes Care* 2014;37(6):1554-62.
7. Cooper MN, McNamara KA, de Klerk NH, Davis EA, Jones TW. School performance in children with type 1 diabetes: A contemporary population-based study. *Pediatric Diabetes* 2016;17(2):101-11.
8. Chowdry H, Crawford C, et al. Widening participation in higher education: analysis using linked administrative data. *Journal of the Royal Statistical Society Series A Statistics in Society* 2013;176(2):431-57.
9. Amillategui B, Calle JR, Alvarez MA, Cardiel MA, Barrio R. Identifying the special needs of children with Type 1 diabetes in the school setting. An overview of parents' perceptions. *Diabetic medicine: a journal of the British Diabetic Association* 2007;24(10):1073-9.
10. Desrocher M, Rovet J. Neurocognitive correlates of type 1 diabetes mellitus in childhood. *Child Neuropsychol* 2004;10:36-52.
11. Gaudieri P, et al. Cognitive function in children with type 1 diabetes: a meta-analysis. *Diabetes Care* 2008;31:1892-7.
12. Naguib J, et al. Neuro-cognitive performance in children with type 1 diabetes- a meta-analysis. *J Pediatr Psychol* 2009;34:271-82.
13. He J, et al. Glycemic extremes are related to cognitive dysfunction in children with type 1 diabetes: A meta-analysis. *J Diabetes Investig* 2018;9(6):1342–53.
14. Diabetes UK. Diabetes in schools - legal information 2017. Available from: https://www.diabetes.org.uk/guide-to-diabetes/your-child-and-diabetes/schools/diabetes-in-schools-legal-information (Accessed 20th August 2018)
15. Her Majesty's Stationery Office (HMSO). Equality Act 2010 (England, Scotland, Wales). 2010. Available from: http://www.legislation.gov.uk/ukpga/2010/15/pdfs/ukpga_20100015_en.pdf.
16. Her Majesty's Stationery Office (HMSO). Children and Families Act 2014 (England). 2014. Available from: http://www.legislation.gov.uk/ukpga/2014/6/pdfs/ukpga_20140006_en.pdf.
17. Her Majesty's Stationery Office (HMSO). Additional Learning Needs and Education Tribunal Act (Wales) 2018. Available from: http://www.legislation.gov.uk/anaw/2018/2/pdfs/anaw_20180002_en.pdf.
18. Her Majesty's Stationery Office (HMSO). Education (Additional Support For Learning) Act 2004 (Scotland). 2004. Available from: https://www.legislation.gov.uk/asp/2004/4/pdfs/asp_20040004_en.pdf.
19. Department of Education and Department of Health Social Services and Public Safety. Supporting Pupils With Medication Needs (Northern Ireland). Available from: *https://www.education-ni.gov.uk/sites/default/files/publications/de/supporting-pupils-with-medical-needs.pdf*. (Accessed 20th August 2018)
20. Oakley N, Kneale D, Mann M, Hilliar M, Tan J, Dayan C, et al. The Association between Type 1 Diabetes Mellitus and Educational Attainment in Childhood: A Systematic Review. PROSPERO International Prospective Register of Systematic Reviews CRD42017084078. Available from: http://www.crd.york.ac.uk/PROSPERO/display_record.php?ID=CRD42017084078. (Accessed 20th August 2018)

For peer review only



For peer review only - http://bmjopen.bmj.com/site/about/guidelines.xhtml

21. Oakley N, Kneale D, Mann M, et al. Association between type 1 diabetes mellitus and educational attainment in childhood: a systematic review protocol. *BMJ Open* 2018;8:e021893.
22. Higgins J, Green S. Cochrane Handbook for Systematic Reviews of Intervention. Version 5.1.0 [updated March 2011]. The Cochrane Collaboration. Available from: http://handbook-5-1.cochrane.org/. (Accessed 20th August 2018)
23. Moher D, Liberati A, Tetzlaff J, Altman D, The PRISMA Group. Preferred Reporting Items for Systematic Reviews and Meta-Analyses: The PRISMA Statement. *PLoS Medicine* 2009;6(7):e1000097.
24. (NIH) U.S. National Library of Medicine. ClinicalTrials.gov. Available from: https://clinicaltrials.gov/. (Accessed 20th August 2018)
25. BioMed Central Ltd. International Standard Randomised Controlled Trial Number (ISRCTN) Registry 2018. Available from: http://www.isrctn.com/. (Accessed 20th August 2018)
26. World Health Organisation. International Clinical Trials Registry Platform (ICTRP). Available from: http://www.who.int/ictrp/en/. (Accessed 20th August 2018)
27. Thomas J, Brunton J, Graziosi S. EPPI-Reviewer 4: software for research synthesis. EPPI-Centre Software. London: Social Science Research Unit, UCL Institute of Education 2010.
28. Schünemann H, Brożek J, Guyatt G, Oxman A (eds). GRADE handbook for grading quality of evidence and strength of recommendations. Updated October 2013: The GRADE Working Group. Available from: www.guidelinedevelopment.org/handbook. (Accessed 20th August 2018)
29. Dahlquist G, Kallen B. School performance in children with type 1 diabetes-a population-based register study. *Diabetologia* 2007;50(5):957-64.
30. Nasuuna E, et al. Examining the relationship between childhood health conditions and health service utilisation at school entry and subsequent academic performance in a large cohort of Australian children. *Journal of Paediatrics and Child Health* 2016;52:750-8.
31. Crump C, Rivera D, London R, Landau M, Erlendson B, Rodriguez E. Chronic health conditions and school performance among children and youth. *Annals of Epidemiology* 2013;23(4):179-84.
32. von Stumm S, et al. Childhood behavior problems and health at midlife: 35-year follow-up of a Scottish birth cohort. *Journal of child psychology and psychiatry and allied disciplines* 2011;52(9):992-1001.
33. Elrayah H, et al. Economic burden on families of childhood type 1 diabetes in urban Sudan. *Diabetes Research and Clinical Practice* 2005;70(2):159-65.
34. Meo SA, Alkahlan MA, Al-Mubarak MA, Al-Obayli MS, Melaibary BA, Bin Dous AN, et al. Impact of type 1 diabetes mellitus on academic performance. *Journal of International Medical Research* 2013;41(3):855-8.
35. National Institute of Clinical Excellence. NICE Guideline [NG18] Diabetes (Type 1 and Type 2) in children and young people: diagnosis and management. Available from: https://www.nice.org.uk/guidance/ng18. (Accessed 20th August 2018)



For peer review only - http://bmjopen.bmj.com/site/about/guidelines.xhtml

# Figure 1

## PRISMA 2009 Flow Diagram

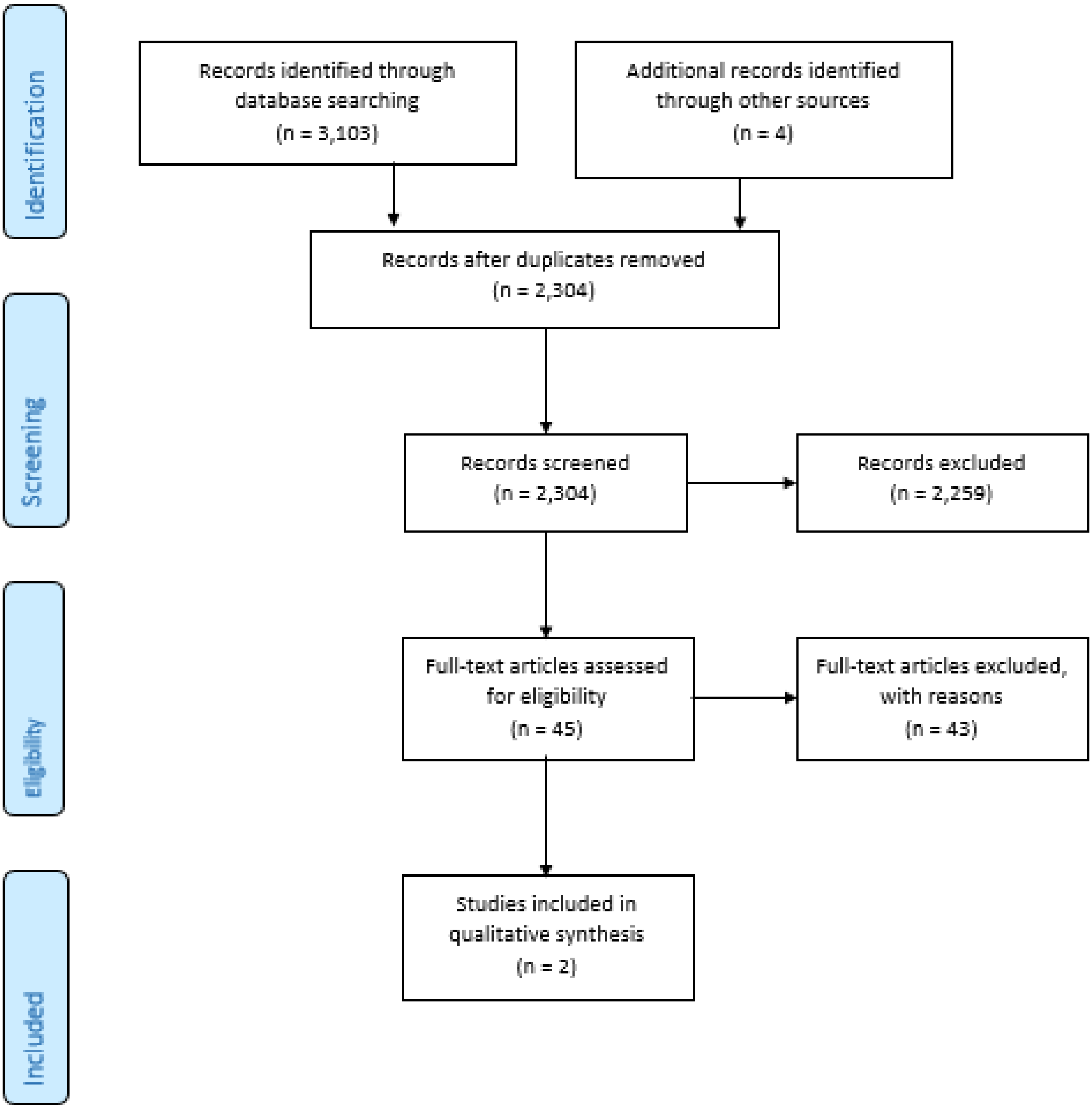


*From:* Moher D, Liberati A, Tetzlaff J, Altman DG, The PRISMA Group (2009). *P*referred *R*eporting *I*tems for *S*ystematic Reviews and *M*eta-*A*nalyses: The PRISMA Statement. PLoS Med 6(7): e1000097. doi:10.1371/journal.pmed1000097

**Appendix 1: Search Strategies**

Search Strategy for Ovid MEDLINE (R) (1946 to present), Ovid MEDLINE(R) Epub Ahead of Print, In-Process & Other Non-Indexed Citations, Ovid EMBASE (1947 to present).

| | Searches |
|---|---|
| 1 | exp Child/ |
| 2 | exp Pediatrics/ |
| 3 | exp Adolescent/ |
| 4 | teen*.ti,ab. |
| 5 | child*.ti,ab. |
| 6 | adolescen*.ti,ab. |
| 7 | p?ediatric*.ti,ab. |
| 8 | juvenile*.ti,ab. |
| 9 | youth*.ti,ab. |
| 10 | (young adj3 (person* or people)).ti,ab. |
| 11 | minors.ti,ab. |
| 12 | or/1-11 |
| 13 | exp Diabetes Mellitus, Type 1/ |
| 14 | (type 1 diabetes or T1D or T1DM or diabet*).ti,ab. |
| 15 | (Insulin adj3 dependent).ti,ab. |
| 16 | 13 or 14 or 15 |
| 17 | (academic* adj3 (attain* or grade* or performance* or success* or status* or outcome* or result* or mark* or achiev* or score* or progress*)).ti,ab. |
| 18 | (educat* adj3 (attain* or grade* or performance* or success* or outcome* or result* or status* or mark* or achiev* or score* or progress*)).ti,ab. |
| 19 | (school* adj3 (attain* or grade* or performance* or success* or status* or outcome* or result* or mark* or achiev* or score* or progress*)).ti,ab. |
| 20 | exp Educational Status/ |
| 21 | or/17-20 |
| 22 | 12 and 16 and 21 |
| 23 | limit 22 to yr="2004 -Current" |

Search Strategy for Thomson Reuters Web of Science

For peer review only

| | Searches |
|---|---|
| #4 | #3 AND #2 AND #1<br>(Timespan=2004-present) |
| #3 | TS=(type 1 diabetes or t1d or t1dm or diabet* or insulin near/3 dependent) |
| #2 | TS=(((academic* or educat* or school*) near/3 (attain* or grade* or performance* or success* or status* or outcome* or result* or mark* or achiev* or score* or progress*))) |
| #1 | TS=(child* or p?ediatric or juvenile* or adolescen* or teen* or minors or juvenile or youth* or (young near/3 (people or person*))) |

For peer review only

Search Strategy for EBSCO British Education Index (BEI), EBSCO Education Resources Information Center (ERIC) & EBSCO Cumulative Index to Nursing and Allied Health Literature (CINAHL)

| | Searches |
|---|---|
| S10 | S3 AND S6 AND S9<br>(Limiter=2004-present) |
| S9 | S7 OR S8 |
| S8 | TI ((academic* or educat* or school*) N3 (attain* or grade* or performance* or success* or status* or outcome* or result* or mark* or achiev* or score* or progress*)) |
| S7 | AB ((academic* or educat* or school*) N3 (attain* or grade* or performance* or success* or status* or outcome* or result* or mark* or achiev* or score* or progress*)) |
| S6 | S4 OR S5 |
| S5 | AB child* or p?ediatric or adolescen* or teen* or minors or juvenile* or youth* or (young N3 (people or person*) |
| S4 | TI child* or p?ediatric or adolescen* or teen* or minors or juvenile* or youth* or (young N3 (people or person*) |
| S3 | S1 OR S2 |
| S2 | AB(diabet* or type 1 diabetes or T1D or T1DM) |
| S1 | TI(diabet* or type 1 diabetes or T1D or T1DM) |

**Appendix 2:**

| **Journal title** |
|---|
| New England Journal of Medicine |
| Lancet |
| JAMA |
| Lancet Internal Medicine |
| BMJ |
| Diabetes Care |
| PLOS Medicine |
| Diabetologia |
| Pediatric Diabetes |
| Endocrine Reviews |
| Nature Reviews Endocrinology |
| Trends in Endocrinology & Metabolism |
| Diabetes |
| Journal of Clinical Endocrinology & Metabolism |
| Diabetes, Obesity & Metabolism |
| American Journal of Physiology – Endocrinology and Metabolism |
| Reviews in Endocrine & Metabolic Disorders |
| Journal of Endocrinology |
| European Journal of Endocrinology |
| Diabetes/ Metabolism Research and Reviews |
| Best Practice and Research in Clinical Endocrinology and Metabolism |
| Diabetes and Metabolism Journal |

**Table 1: List of key journals for which electronic table of contents were searched for additional relevant articles.**

For peer review only

**Appendix 3:**

| **Excluded Studies** |
|---|
| Abusrewil S. (2013) Epidemiology of childhood diabetes in western part of Libya (1989-2013). Pediatric Diabetes 18:117. |
| Almqvist C et al. (2016) Sibship and risk of asthma in a total population: A disease comparative approach. Journal of Allergy and Clinical Immunology 138(4):1219-1222e3. |
| Bass L (2011) Social Focus on Health and Children's Well-being. Sociological Inquiry 81: 495-498 |
| Bezerra A et al. (2012) School attainment in childhood is an independent risk factor of dementia in late life: results from a Brazilian sample. International Psychogeriatrics 24(1): 55-61. |
| Bjerkedal T et al. (2006) A follow up of persons who received basic and/or supplemental benefits in childhood. Tidsskr Nor Legeforen 126: 436-439 |
| Bortes C et al. (2018) Health problems during childhood and school achievement: Exploring associations between hospitalization exposures, gender, timing, and compulsory school grades. PLoS ONE 13(12):e0208116. |
| Catalano D et al. (2004) Journal of Allergy and Clinical Immunology. 155(5): 175-8. |
| Champaloux S, Young D (2015) Childhood Chronic Health Conditions and Educational Attainment: A Social Ecological Approach. Journal of Adolescent Health 56: 98-105 |
| Chaudhry T, Klonoff D (2013) SMBG out of control: the need for educating patients about control solution. Diabetes Educator 39: 689-695 |
| Compas B, et al. (2017) Neurocognitive Deficits in Children With Chronic Health Conditions. American Psychologist 72: 326-338 |
| Cooper MN, McNamara KA, de Klerk NH, Davis EA, Jones TW (2016) School performance in children with type 1 diabetes: A contemporary population-based study. Pediatric Diabetes 17: 101-111. |
| Crump C, Rivera D, London R, Landau M, Erlendson B, Rodriguez E (2013) Chronic health conditions and school performance among children and youth. Annals of Epidemiology 23: 179-184. |
| Elrayah H, et al. (2005) Economic burden on families of childhood type 1 diabetes in urban Sudan. Diabetes Research and Clinical Practice 70: 159-165 |
| Engelke M et al. (2008) School nurse case management for children with chronic illness: health, academic, and quality of life outcomes. Journal of School Nursing 24(4):205-214 |
| Erkolahti R, Ilonen T (2005) Academic Achievement and the Self-Image of Adolescents with Diabetes Mellitus Type-1 And Rheumatoid Arthritis. Journal of Youth and Adolescence 34: 199. |
| Fraser A et al. (2012) Associations of existing diabetes, gestational diabetes, and glycosuria with offspring IQ and educational attainment: The avon longitudinal study of parents and children. Experimental Diabetes Research 963735. |
| Gonzalez A, et al. (2012) Childhood and family influences on depression, chronic physical conditions, and their comorbidity: Findings from the Ontario Child Health Study. Journal of Psychiatric Research 46: 1475-1482 |
| Hassan M et al. (2017) Assessment of health-related quality of life in Egyptian adolescents with type 1 diabetes: DEMPU survey. Journal of Pediatric Endocrinology and Metabolism 30(3): 277-283. |
| Jameson P et al. (2006) Diabetes, cognitive function, and school performance. School Nurse News 23(3):34-36. |
| Jesic M et al. (2013) School problems in children and adolescents with type 1 diabetes. Pediatric Diabetes 18:102-103. |
| Lansing A et al. (2018) Academic achievement and metabolic control in adolescents with type 1 diabetes, Children's Health Care 47(1):16-33 |
| Liaqat A at al. (2017) Epidemology of type-I diabetes and its economic burden on society. Pakistan Journal of Medical and Health Sciences 11(2):714-717. |
| Lynch P et al. (2004) Predicting academic achievement in youths with type 1 diabetes: A biopsychosocial model. Diabetes 53:A436. |
| Maslow G et al. (2011) Growing up with a chronic illness: social success, educational/vocational distress. Journal of Adolescent Health 49(2):206-12. |
| Maslow G et al. (2012) Protective connections and educational attainment among young adults with childhood-onset chronic illness. 82(8):364-70. |

For peer review only

| |
|---|
| Meo SA, Alkahlan MA, Al-Mubarak MA, et al. (2013) Impact of type 1 diabetes mellitus on academic performance. Journal of International Medical Research 41: 855-858 |
| Merrick H et al. (2015) Characteristics of young people with long term conditions close to transfer to adult health services. BMC health services research 15:435. |
| Milovanovic I et al. (2012) Entred-Ado study: Health, education and risk behaviours of adolescents with diabetes. Diabetologia 1:S140. |
| Mitosi N et al. (2013) Family role in treatment adherence of type 1 diabetes: Research data from Greek population. Pediatric Diabetes 18:110. |
| Nasuuna E, Santoro G, Kremer P, de Silva AM (2016) Examining the relationship between childhood health conditions and health service utilisation at school entry and subsequent academic performance in a large cohort of Australian children. Journal of Paediatrics and Child Health 52: 750-758 |
| Neves C et al. (2013) Obesity, diet and physical activity: The reality of our teenagers. Atencion Primaria 2:84. |
| Nielson H et al. (2016) Type 1 diabetes, quality of life, occupational status and education level - A comparative population-based study. Diabetes Research & Clinical Practice 121:62-68 |
| Ovesen L et al. (2015) Education level, occupational status and quality of life in adults with type 1 diabetes and in the general population: A comparative study. Diabetologia 1:S412. |
| Roman R et al. (2016) School performance in children and adolescents with type 1 diabetes. Hormone Research in Paediatrics 86:51. |
| Roman R et al. (2017) Metabolic control and school performance in children with type 1 diabetes. Revista Chilena de Pediatria 88(5):586-594. |
| Ryan C. (2012) Does severe hypoglycaemia disrupt academic achievement in children with early onset diabetes? Developmental Medicine and Child Neurology 54(5):393-394 |
| Schiel R et al. (2016) Plasma copeptin in children and adolescents with type 1 diabetes mellitus in comparison to healthy controls. Diabetes Research and Clinical Practice 118:156-161. |
| Steen Carlsson K et al. (2015) Type 1 diabetes with early onset and educational field at upper secondary and university level: Is own experience an asset for a health care carrier? Diabetologia 1:S411. |
| Tahirovic H et al. (2013) Impact of socioeconomic status on health-related quality of life in children with type 1 diabetes mellitus in Bosnia and Herzegovina. Minerva Pediatrica 65(2):207-212. |
| Taras H et al. (2005) Chronic health conditions and student performance at school. Journal of School Health 75(7):255-266. |
| von Stumm S et al. (2011) Childhood behavior problems and health at midlife: 35-year follow-up of a Scottish birth cohort. Journal of child psychology and psychiatry and allied disciplines 52(9):992-1001. |
| Wennick A et al. (2011) Attained education and self-assessed health later in life when diagnosed with diabetes in childhood: a population-based study. Pediatric Diabetes 12(7):619-26. |
| Winnick J et al. (2017) Metabolic Control and Academic Achievement Over Time Among Adolescents With Type 1 Diabetes. School Psychology Quarterly 32(1):105-117. |

**Table 1: List of excluded studies following full text review.**

For peer review only

**Appendix 4:**

| | Newcastle Ottawa Scale | | | | Overall Risk of Bias |
|---|---|---|---|---|---|
| **Study** | **Selection** | **Comparability** | **Outcome** | **Overall** | |
| **Dahlquist & Kallen (D2007)** | 4/4 | 2/2 | 2/3 | **8/9** | **Low** |
| **Persson et al. (D2013)** | 4/4 | 2/2 | 3/3 | **9/9** | **Low** |

**Table 1: Newcastle Ottawa Scale ratings for included studies.**

For peer review only

# PRISMA 2009 Checklist

| Section/topic | # | Checklist item | Reported on page # |
|---|---|---|---|
| **TITLE** | | | |
| Title | 1 | Identify the report as a systematic review, meta-analysis, or both. | 1 |
| **ABSTRACT** | | | |
| Structured summary | 2 | Provide a structured summary including, as applicable: background; objectives; data sources; study eligibility criteria, participants, and interventions; study appraisal and synthesis methods; results; limitations; conclusions and implications of key findings; systematic review registration number. | 2 |
| **INTRODUCTION** | | | |
| Rationale | 3 | Describe the rationale for the review in the context of what is already known. | 3, 4 |
| Objectives | 4 | Provide an explicit statement of questions being addressed with reference to participants, interventions, comparisons, outcomes, and study design (PICOS). | 5 |
| **METHODS** | | | |
| Protocol and registration | 5 | Indicate if a review protocol exists, if and where it can be accessed (e.g., Web address), and, if available, provide registration information including registration number. | 2, 5 |
| Eligibility criteria | 6 | Specify study characteristics (e.g., PICOS, length of follow-up) and report characteristics (e.g., years considered, language, publication status) used as criteria for eligibility, giving rationale. | 5, 6, Protocol |
| Information sources | 7 | Describe all information sources (e.g., databases with dates of coverage, contact with study authors to identify additional studies) in the search and date last searched. | 5, 6, Appendix 1+2 |
| Search | 8 | Present full electronic search strategy for at least one database, including any limits used, such that it could be repeated. | 5, Appendix 1 |
| Study selection | 9 | State the process for selecting studies (i.e., screening, eligibility, included in systematic review, and, if applicable, included in the meta-analysis). | 5, Protocol |
| Data collection process | 10 | Describe method of data extraction from reports (e.g., piloted forms, independently, in duplicate) and any processes for obtaining and confirming data from investigators. | 5, 6, Protocol |
| Data items | 11 | List and define all variables for which data were sought (e.g., PICOS, funding sources) and any assumptions and simplifications made. | Protocol |
| Risk of bias in individual studies | 12 | Describe methods used for assessing risk of bias of individual studies (including specification of whether this was done at the study or outcome level), and how this information is to be used in any data synthesis. | 5, 6 |
| Summary measures | 13 | State the principal summary measures (e.g., risk ratio, difference in means). | 6 |
| Synthesis of results | 14 | Describe the methods of handling data and combining results of studies, if done, including measures of consistency (e.g., $I^2$) for each meta-analysis. | 6 |

For peer review only - http://bmjopen.bmj.com/site/about/guidelines.xhtml

# PRISMA 2009 Checklist



| Section/topic | # | Checklist item | Reported on page # |
|---|---|---|---|
| Risk of bias across studies | 15 | Specify any assessment of risk of bias that may affect the cumulative evidence (e.g., publication bias, selective reporting within studies). | 5, 6 |
| Additional analyses | 16 | Describe methods of additional analyses (e.g., sensitivity or subgroup analyses, meta-regression), if done, indicating which were pre-specified. | 6 |
| **RESULTS** | | | |
| Study selection | 17 | Give numbers of studies screened, assessed for eligibility, and included in the review, with reasons for exclusions at each stage, ideally with a flow diagram. | 6, 7, Figure 1, Table 1, Appendix 3 |
| Study characteristics | 18 | For each study, present characteristics for which data were extracted (e.g., study size, PICOS, follow-up period) and provide the citations. | 7, 8, 14, 15, Table 2. |
| Risk of bias within studies | 19 | Present data on risk of bias of each study and, if available, any outcome level assessment (see item 12). | 8-10, Table 4, Appendix 4. |
| Results of individual studies | 20 | For all outcomes considered (benefits or harms), present, for each study: (a) simple summary data for each intervention group (b) effect estimates and confidence intervals, ideally with a forest plot. | 6-10, Tables 3+4. |
| Synthesis of results | 21 | Present results of each meta-analysis done, including confidence intervals and measures of consistency. | N/A |
| Risk of bias across studies | 22 | Present results of any assessment of risk of bias across studies (see Item 15). | 6-10, Table 4, Appendix 4 |
| Additional analysis | 23 | Give results of additional analyses, if done (e.g., sensitivity or subgroup analyses, meta-regression [see Item 16]). | 6-10, Table 3 |
| **DISCUSSION** | | | |
| Summary of evidence | 24 | Summarize the main findings including the strength of evidence for each main outcome; consider their relevance to key groups (e.g., healthcare providers, users, and policy makers). | 8-10, Tables 3+4 |
| Limitations | 25 | Discuss limitations at study and outcome level (e.g., risk of bias), and at review-level (e.g., incomplete retrieval of identified research, reporting bias). | 10-12 |
| Conclusions | 26 | Provide a general interpretation of the results in the context of other evidence, and implications for future research. | 10-12 |
| **FUNDING** | | | |
| Funding | 27 | Describe sources of funding for the systematic review and other support (e.g., supply of data); role of funders for the systematic review. | 13 |

*From:* Moher D, Liberati A, Tetzlaff J, Altman DG, The PRISMA Group (2009). Preferred Reporting Items for Systematic Reviews and Meta-Analyses: The PRISMA Statement. PLoS Med 6(7): e1000097.

For peer review only - http://bmjopen.bmj.com/site/about/guidelines.xhtml

# PRISMA 2009 Checklist

doi:10.1371/journal.pmed1000097
For more information, visit: www.prisma-statement.org.


For peer review only

BMJ Open

# Type 1 Diabetes Mellitus and Educational Attainment in Childhood: A Systematic Review



| | |
|---|---|
| Journal: | *BMJ Open* |
| Manuscript ID | bmjopen-2019-033215.R1 |
| Article Type: | Original research |
| Date Submitted by the Author: | 19-Nov-2019 |
| Complete List of Authors: | Oakley, Natalie; Cardiff University School of Medicine, Division of Population Medicine<br>Kneale, Dylan; UCL Institute of Education,<br>Mann, Mala; Cardiff University, University Library Services<br>Hilliar, Mariann; University Hospital of Wales, Cardiff University Library<br>Dayan, Colin; Cardiff University School of Medicine<br>Gregory, John; Cardiff University School of Medicine, Division of Population Medicine<br>French, Robert; Cardiff University School of Medicine |
| <b>Primary Subject Heading</b>: | Diabetes and endocrinology |
| Secondary Subject Heading: | Paediatrics |
| Keywords: | DIABETES & ENDOCRINOLOGY, Paediatric endocrinology < DIABETES & ENDOCRINOLOGY, Community child health < PAEDIATRICS |
| | |



SCHOLARONE™
Manuscripts

# Type 1 Diabetes Mellitus and Educational Attainment in Childhood: A Systematic Review

Oakley N[1], Kneale D[2], Mann M[3], Hilliar M[4], Dayan C[5], Gregory JW[1], French R*[5]

*Corresponding author: Dr Robert French. Email: frenchr3@cardiff.ac.uk

[1]Division of Population Medicine, Cardiff University School of Medicine, Neuadd Meirionnydd, University Hospital Wales, Cardiff, CF14 4YS.

[2] Social Science Research Unit (SSRU), University College London, 18 Woburn Street, London, WC1H 0NR

[3]Specialist Unit for Review Evidence (SURE), Cardiff University School of Medicine, Neuadd Meirionnydd, University Hospital Wales, Cardiff, CF14 4YS.

[4] Cardiff University Library, Cochrane Building, University Hospital Wales, Heath Park, CF14 4YU.

[5] Cardiff University School of Medicine, University Hospital Wales, Heath Park, Cardiff, CF14 4YS.

Word count: 3970

For peer review only



For peer review only - http://bmjopen.bmj.com/site/about/guidelines.xhtml

# ABSTRACT

**Objectives**

The primary objective of this systematic review was to evaluate available literature on whether type 1 diabetes mellitus (T1DM) has an impact on educational attainment in individuals undertaking high stakes standardised testing at the end of compulsory schooling.

**Design**

A systematic review was undertaken comparing educational attainment for individuals with and without T1DM who have undertaken high stakes testing at the end of compulsory schooling.

**Data sources**

A comprehensive search of MEDLINE, MEDLINE (Epub Ahead of Print, In-Process & Other Non-Indexed Citations), EMBASE, Web of Science, British Education Index (BEI), Education Resources Information Center (ERIC) and Cumulative Index to Nursing and Allied Health Literature (CINAHL) was undertaken on 15$^{th}$ January 2018 and updated on 17$^{th}$ January 2019.

**Eligibility Criteria**

Included studies fulfilled the following criteria: observational study or randomised controlled trial; included individuals who have undertaken high stakes testing at the end of compulsory schooling; and compared the grades obtained by individuals with T1DM with a representative population control.

**Data extraction and synthesis**

Two reviewers performed study selection and data extraction independently. Quality and risk of bias in the observational studies included were assessed using the Newcastle-Ottawa Scale. A detailed narrative synthesis of the included studies was completed.

**Results**

3,103 articles were identified from the database search, with two Swedish cohort studies (using the same linked administrative data) meeting final inclusion criteria. A small but statistically significant difference was reported in mean final grades, with children with T1DM found to have lower mean grades than their non-diabetic counterparts (adjusted mean difference 0.07-0.08).

**Conclusions**

More contemporary research is required to evaluate the impact of T1DM in childhood on educational attainment in individuals undertaking high stakes standardised testing at the end of compulsory schooling, taking into consideration the substantial advances in management of T1DM in the last decade.


**Keywords**

Diabetes mellitus type 1; Educational status; Achievement; Adolescent; Child; School; Systematic Review

**PROSPERO Registration number**

CRD42017084078



For peer review only - http://bmjopen.bmj.com/site/about/guidelines.xhtml

## STRENGTHS AND LIMITATIONS OF THIS STUDY

- Previous studies have found an association between T1DM in childhood and reduced cognitive function, however there is less evidence assessing the effect of T1DM on educational attainment, particularly in the form of high stakes examinations.
- This review has comprehensively evaluated available literature reporting the impact of T1DM on educational attainment in individuals undertaking high stakes standardised testing at the end of compulsory schooling.
- The findings were reported using the Cochrane Handbook for Systematic Reviews of Interventions and recommended methods and checklist of the Preferred Reporting Items for Systematic Reviews and Meta-Analyses (PRISMA), with the quality of evidence assessed using the Grading of Recommendations Assessment, Development and Evaluation (GRADE) system.
- Study selection and data extraction were performed independently by two reviewers, and the quality and risk of bias of the studies included in this review were assessed using the Newcastle-Ottawa Scale (NOS).
- Limitations of this review include the use of narrow inclusion thresholds, commensurate with the research objectives, resulting in a small number of included studies thereby precluding the use of meta-analysis and limiting generalisability of the results.

For peer review only



For peer review only - http://bmjopen.bmj.com/site/about/guidelines.xhtml

# INTRODUCTION

## Background & Rationale

Type 1 diabetes mellitus (T1DM) is an autoimmune disorder which destroys the insulin-producing beta cells in the pancreas, preventing the body from adequately regulating blood glucose levels.(1) According to the 2017 International Diabetes Federation (IDF) Diabetes Atlas 8th edition, the number of children diagnosed with type 1 diabetes is increasing annually, with an estimated 96,100 new cases worldwide every year.(2)

Physiological complications of diabetes can include hypoglycaemia, hyperglycaemia & ketoacidosis.(1) These complications, especially if recurrent, have the potential to impact on educational attainment via a combination of mechanisms including altered cognitive function and non-attendance for acute treatment.(3, 4) Glucose is the principal energy source of the human brain. Hypoglycaemia, particularly if severe or recurrent, can result in neuroglycopenia which, in turn, may result in neuronal injury and subsequent cognitive impairment.(5) Hyperglycaemia, particularly when associated with ketoacidosis, has also been found to have detrimental effects on the brain via damage to white matter, disrupted functioning of the blood-brain barrier and transient focal cerebral oedema. These changes can again impair cerebral functioning resulting in cognitive impairment.(6) There is also the potential for attendance and attainment to be affected by hospital appointments or illness at critical points in the educational trajectory.(7) This is especially important when considering high stakes testing at the end of compulsory schooling which has the potential to significantly impact future social and economic outcomes, such as social status, employment and income(8).

Broadly, high stakes testing can be defined as examinations or assessments which carry serious and important consequences for the individuals taking the tests. In the case of high stakes tests at the end of compulsory schooling, these important consequences may include subsequent educational and employment prospects and opportunities. High stakes tests are often 'standardised', meaning there are specific rules and regulations involved in providing and completing the test. This aims to ensure every individual taking the test receives the same resources and directions, allowing a more accurate interpretation of individuals' performance and ability.(9)

Many patients and their families report worries about the support available for children with diabetes at school and concerns about the potential negative impact that T1DM may have on school attendance.(4, 7) In an observational study published in 2007, Amillategui et al. utilised self-reporting questionnaires to identify specific parental concerns regarding T1DM in the school setting. Many of these concerns focused on aspects such as glycaemic control, social integration, medication administration and understanding of staff members, which may affect children's attainment, wellbeing and experience in school.(10)

Previous literature has studied the effects of T1DM on cognitive functioning in children. In a review published in 2004, Desrocher et al. found an association between T1DM in childhood and poorer neurocognitive outcomes, with the most significant deficits found to be related to younger age of disease onset, episodes of hypoglycaemia and hyperglycaemia around the time of puberty.(11) In



For peer review only - http://bmjopen.bmj.com/site/about/guidelines.xhtml

2008, Gaudieri et al reported that T1DM in childhood was associated with poorer performance in learning and reduced memory skills.(12) Further studies published by Naguib et al. in 2008(13) and He et al. in 2018(14) reported similar findings, suggesting a detrimental impact of T1DM in childhood on cognitive function. However, there is less evidence as to the effect T1DM has on educational attainment, particularly in the form of high stakes examinations, and the magnitude of this impact.(7)

Each of the four home nations within the United Kingdom (England, Northern Ireland, Scotland and Wales) has specific legislation focused on support for children and young people with additional learning needs and medical conditions including T1DM in school.(15-20) Quantifying the impact of T1DM on educational attainment at the end of compulsory schooling and identifying domains most affected may be useful in assessing what and how much support should be focused on children with T1DM in school.

The primary objective of this review was to identify and analyse the available literature on whether T1DM has an impact on educational attainment in individuals undertaking high stakes standardised testing at the end of compulsory schooling.
Secondary objectives included assessment of the effect of T1DM on school attendance and educational attainment at other stages on the educational trajectory if reported.

# METHODS

The protocol for this systematic review is registered with PROSPERO (International Prospective Register of Systematic Reviews) at the NHS Centre for Reviews and Dissemination (CRD) at the University of York (Registration number CRD42017084078)(21) and has been previously published.(22) The Cochrane Handbook for Systematic Reviews of Interventions(23) and recommended methods and checklist of the Preferred Reporting Items for Systematic Reviews and Meta-Analyses (PRISMA)(24) were used to structure this systematic review.

Studies were included if they fulfilled the following inclusion criteria: 1) observational study (including prospective and retrospective cohort and case-control studies) or randomised controlled trial; 2) included individuals who have undertaken high stakes testing at the end of compulsory schooling; and 3) compared the grades obtained by individuals with type 1 diabetes with a representative population control. The primary outcome assessed was grade obtained following high stakes testing at the end of compulsory schooling. Secondary outcomes were school attendance and grades obtained at other stages on the educational trajectory.

Comprehensive electronic literature search strategies were developed to search the following seven databases: Ovid MEDLINE (1946 to present), Ovid MEDLINE (Epub Ahead of Print, In-Process & Other Non-Indexed Citations), Ovid EMBASE (1947 to present), Thomson Reuters Web of Science, EBSCO British Education Index (BEI), EBSCO Education Resources Information Center (ERIC) and EBSCO Cumulative Index to Nursing and Allied Health Literature (CINAHL). Only studies published since January 2004 in the English language were considered. The initial search was undertaken on 15$^{th}$ January 2018 and then updated on 17$^{th}$ January 2019 to identify any further studies. The results for



For peer review only - http://bmjopen.bmj.com/site/about/guidelines.xhtml

the initial and updated searches were combined. The search strategies used for the above databases are given in Appendix 1.

To identify (i) studies in progress, (ii) unpublished research, or (iii) research reported in the grey literature, the electronic Table of Contents (eTOC) of key journals (listed in Appendix 2) and trial registries (25-27) were searched for studies published within the last two years. Finally, additional searches for studies were undertaken via relevant websites including Diabetes.org.uk and jdrf.org.uk, review articles, reference lists and citation tracking of included studies.

After removal of duplicates, two independent reviewers (NO & RF) screened study titles and abstracts using Eppi-Reviewer 4.0.(28) Full texts of potentially eligible records were then screened according to set inclusion criteria. The rationale for the exclusion of studies at each stage was documented. The remaining included studies underwent data extraction by NO & RF independently.
The quality and risk of bias in the observational studies included in this review were assessed by NO & RF independently using the Newcastle-Ottawa Scale (NOS).

The principle outcome measure used in included studies was the 'mean difference' between school grades attained. When assessing the likelihood of achieving different grades, the principle binary outcome measure used was the 'odds ratio'. Meta-analysis was not possible because of the substantial duplication of data linkage from the databases, with both papers including a cohort of participants born 1973 to 1978. As a result, a detailed narrative synthesis of the included papers was undertaken.

The quality of evidence for the primary outcome was assessed using the Grading of Recommendations Assessment, Development and Evaluation (GRADE) system.(29) Results are presented in a 'Summary of findings' table as recommended by the Cochrane Handbook for Systematic Reviews of Interventions.(23)

**Patient and Public Involvement**
Patients and the public were not involved in the development and completion of this systematic review.

# RESULTS

The database search identified 3,103 papers. After duplicates were removed, 2,304 articles underwent abstract screening (Figure 1). A total of 45 papers were shortlisted for full-text review. No full text was available for nine studies, and further information was required for one study. The authors of these studies were contacted, but as no response or clarification was obtained, these studies were excluded.

Table 1 lists the reasons for exclusion for the studies rejected on full-text review (full citations listed in Appendix 3). The abstracts of four additional studies identified from the reference lists of the studies shortlisted for full-text review were screened and rejected.



For peer review only - http://bmjopen.bmj.com/site/about/guidelines.xhtml

**Table 1: Primary reasons for exclusion of studies following full-text review**

| Reason for exclusion | | | | | |
|---|---|---|---|---|---|
| **No final school-based grades assessed** | **No full text available** | **Study not specifically assessing T1DM** | **Not assessing grades following high stakes tests at end of compulsory schooling** | **No comparison between children with and without T1DM** | **Study design not in scope** |
| Bass L. 2011<br>Bjerkedal T et al. 2006<br>Champalaoux S et al. 2015<br>Chaudhry T et al. 2013<br>Compas B et al. 2017<br>Elrayah H et al. 2005<br>Gonzalez A et al. 2012<br>Hassan M et al. 2017<br>Liaqat A at al. 2017<br>Maslow G et al. 2011<br>Maslow G et al. 2012<br>Merrick H et al. 2015<br>Neves C et al. 2013<br>Nielson H et al. 2016<br>Ovesen L et al. 2015<br>Ryan C. 2012<br>Schiel R et al. 2016<br>von Stumm S et al. 2011<br>Wennick A et al. 2011 | Abusrewil S. 2013<br>Catalano D et al. 2004<br>Jesic M et al. 2013<br>Lynch P et al. 2004<br>Milovanovic I et al. 2012<br>Mitosi N et al. 2013<br>Roman R et al. 2016<br>Steen Carlsson K et al. 2015<br>Tahirovic H et al. 2013 | Almqvist C et al. 2016<br>Bezerra A et al. 2012 | Cooper M et al. 2016<br>Crump C et al. 2013<br>Erkolahti R et al. 2005<br>Lansing A et al. 2018<br>Meo S et al. 2013<br>Nasuuna E et al. 2016<br>Roman R et al. 2017 | Engelke M et al. 2008<br>Winnick J et al. 2011<br>Bortes C et al. 2018 | Fraser A et al. 2012<br>Jameson P et al. 2006<br>Taras H et al. 2005 |

Two papers met all of the inclusion criteria, both electronic population cohort studies from Sweden (3, 30), as detailed in Table 2 below. The most recent sought to improve on the analysis of the earlier paper by (i) using different methods, (ii) using more refined model specifications and (iii) adding later life outcomes. Both papers compared mean attainment on a five-point scale (1-5) at age 16 years for those with diabetes to those without diabetes. The sample size decreased for the later study because they used more restricted birth cohorts to allow comparisons of labour force outcomes aged 29 years.

**Table 2: Characteristics of included observational studies.**

| Author (year) | Design | Country | No. cases (known T1DM) | No. controls (no T1DM) | Matching completed (Y/N) | Controlled confounders |
|---|---|---|---|---|---|---|
| Persson et al. (2013) | Electronic population cohort | Sweden | 2,392 | 9,563 | Y | Year of birth, Gender, School year, Level of parental education, Parental income, Parental country of origin |
| Dahlquist and Kallen (2007) | Electronic population cohort | Sweden | 5,159 | 1,330,968 | N | Year of birth, Gender, Maternal age, parity and educational level |

For peer review only



For peer review only - http://bmjopen.bmj.com/site/about/guidelines.xhtml

The primary outcome in these two studies was mean final grade across all school subjects, from compulsory secondary school (Table 3). Persson stated that these grades were teacher-rated, therefore potentially introducing bias if teachers subconsciously adjusted grades because of diabetes status. For the later exam years (1998-2003) used by Dahlquist, the outcome scale was changed and estimates for this period were given separately by subject only. Both papers showed a similar negative effect of diabetes on mean attainment, after adjusting for confounders. Dahlquist estimated children with T1DM achieved 0.08 fewer final mean marks, while Persson estimated children with T1DM achieved 0.07 fewer final mean marks. Persson also used quantile regression to show that the effect of diabetes was strongest in the lowest quantile of attainment.

**Table 3: Effect of T1DM on mean final grade of 1) all school subjects and 2) specific subjects (Maths, Swedish, English, and Sports) at the end of compulsory school.**

| | **Dahlquist and Kallen** | **Persson et al.** |
|---|---|---|
| 1) Mean final grade of all school subjects from compulsory schooling at age 16 (Mean final grade) | T1DM:<br>3.15 (adjusted)<br>No T1DM:<br>3.23 (adjusted)<br>Mean difference (adjusted): 0.08<br><br>T1DM found to have negative impact on mean grade attainment of all school subjects. | T1DM:<br>3.13 +/- 0.75 (unadjusted)<br>No T1DM:<br>3.21 +/- 0.72 (unadjusted)<br>Mean difference (adjusted): 0.07 +/- 0.02, p<0.001.<br>T1DM found to have negative impact on mean grade attainment of all school subjects. |
| Additional statistical analysis performed | Mean t value -0.24 +/- 0.04<br>t* value -5.19 p<0.001 | Quantile regression - Negative effect of T1DM greatest in the lowest quantile of attainment, diminishing with increasing attainment quantiles, becoming statistically insignificant for the highest attaining quantile. |
| 2) Mean final grade of specific subjects (Maths, Swedish, English and Sports) from compulsory school at age 16 | Odds ratios used to compare attainment of each grade in specific subjects in order to estimate effect of T1DM.<br>• Sports/Athletics: Clear negative association seen between T1DM & grade attainment.<br>• English: Similar pattern to Sports/Athletics however weaker association.<br>• Maths and Swedish: Children with T1DM found to be more likely to achieve lower grades however no clear differences were seen for high grades. | Mean difference in attained grades (unadjusted), predicted probability of achieving grades 1-5 in specific subjects, and odds ratios using ordered logistic regression used to estimate effect of T1DM.<br>• Sports/Athletics: Clear negative association seen between T1DM & grade attainment.<br>• Similar trend seen in all subjects - largest differences in attainment seen in Sports/Athletics, smallest differences seen in Maths. |

Both studies also analysed the mean attainment score in four core subjects- Maths, Swedish, English and Sports. In Sports/Athletics, children with T1DM were found to be more likely to achieve lower grades and less likely to achieve higher grades. The quality of evidence for these outcomes, assessed using the Grading of Recommendations Assessment, Development and Evaluation (GRADE) system(29), is presented in the 'Summary of findings' table (Table 4).



For peer review only - http://bmjopen.bmj.com/site/about/guidelines.xhtml

## Table 4: Summary of Findings Table

### The Association between Type 1 Diabetes Mellitus and Educational Attainment in Childhood

**Patient or population**: Individuals who have undertaken high stakes testing at the end of compulsory schooling, under 18 years of age.
**Setting**: School
**Intervention**: Known diagnosis of T1DM.
**Comparison**: No known diagnosis of T1DM.

| Outcomes | Impact | № of participants (studies) | The certainty of the evidence (GRADE) |
|---|---|---|---|
| Mean final grade of all school subjects from compulsory schooling at age 16 (Mean final grade) | Both papers found significantly lower attainment of between 0.07-0.08 marks achieved by children with T1DM compared with their non-diabetic counterparts. | (2 observational studies) | ⊕◯◯◯ VERY LOW [a,b,c,d] |
| Mean final grade of specific subjects (Maths, Swedish, English and Sports) from compulsory school at age 16 | **Dahlquist and Kallen** tested for differences in the odds for each level of attainment by subject in the raw categories and in an alternative four categories where the raw numeric scores (1988-97) were manipulated to have the same number of categories as the alphabetical marks (1998-2003). In Sports, children with T1DM had a greater odds of a low score and a lower odds of a high score. In Maths and Swedish, children with T1DM had a higher odds of a low score (no clear differences for high scores), and there were no clear effects for English.<br><br>**Persson et al** showed a similar pattern, though all the comparisons of levels within subjects were statistically significant (though comparison for the mean attainment scores for Maths was not significant), with the largest differences in attainment for Sports and the smallest differences for Maths. | (2 observational studies) | ⊕◯◯◯ VERY LOW [a,b,c,d] |

**GRADE Working Group grades of evidence**
**High certainty:** We are very confident that the true effect lies close to that of the estimate of the effect
**Moderate certainty:** We are moderately confident in the effect estimate: The true effect is likely to be close to the estimate of the effect, but there is a possibility that it is substantially different
**Low certainty:** Our confidence in the effect estimate is limited: The true effect may be substantially different from the estimate of the effect
**Very low certainty:** We have very little confidence in the effect estimate: The true effect is likely to be substantially different from the estimate of effect

*Explanations*
a. Both included studies control for gender, academic year and maternal education. Dahlquist and Kallen additionally condition on maternal age and parity, while Persson et al additionally control for paternal education, long-term parental income and parental country of origin.
b. Heterogeneity could not be reliably assessed as only two papers met final inclusion criteria (1 study with updated methodology). As a result, there is a substantial amount of duplication of cases from the databases, with both papers including a cohort of participants born 1973 to 1978.
c. Publication bias could not be reliably assessed as only two papers met final inclusion criteria (one study with updated methodology).



For peer review only - http://bmjopen.bmj.com/site/about/guidelines.xhtml

d. Total no. of participants unable to be combined because of the unknown quantity of data duplication from the same cohort of participants born 1973 to 1978. (With T1DM – Dahlquist N=5,159 & Persson N=2,392. Without T1DM – Dahlquist N=1,330,968, Persson N=9,563).

For peer review only



For peer review only - http://bmjopen.bmj.com/site/about/guidelines.xhtml

Both studies estimated the effect of age of diagnosis using further models, suggesting lower grade attainment with earlier diagnosis. Dahlquist estimated conditional means for age groups of diagnosis (<2, 2-5, 5-10, 10-15 years), reporting the lowest mean grades in children diagnosed before two years of age (2.97), with means of 3.08 to 3.17 in categories of older age at diagnoses. However the differences between age groups was found to be not significant. Persson used those without diabetes as the reference group, showing a negative association between earlier diagnosis and attainment. Those diagnosed aged 10-15 years were found to have 0.06 lower attainment, those diagnosed aged 5-9 years were found to have 0.07 lower attainment, and those diagnosed aged 0-4 years were found to have 0.15 lower attainment.

The quality and risk of bias for the included studies was assessed as high-quality methodology with low risk of bias using the Newcastle-Ottawa Scale (NOS). Full NOS ratings can be seen in Appendix 4. Persson scored full marks across the three domains. Dahlquist lost one mark on the outcome assessment domain for not reporting the numbers lost due to linkage (although they did consider the reasons why cases may not be included). Despite both papers being judged to be methodologically sound overall, the certainty of the evidence was deemed to be very low, mainly reflecting the small number of studies identified and available for analysis.

# DISCUSSION

**Summary of evidence**

This systematic review identified two papers (using overlapping Swedish administrative cohorts) assessing the association between type 1 diabetes mellitus in childhood and educational attainment following high stakes testing at the end of compulsory schooling. Both papers found statistically significant lower mean attainment of between 0.07-0.08 marks in those with T1DM. Persson checked this relationship by attainment quantile and showed that the differences were greater for lower attainment levels. Both papers considered within-subject attainment and found a negative effect of T1DM. This effect was particularly pronounced in Sports/Athletics but less clear in Maths. It is of great concern that the largest negative association was seen in Sports/Athletics because exercise is a key tool for managing blood sugar and suggests other potential confounding factors such as parental or teacher anxiety regarding the involvement of diabetic children in physical education at school and the risk of hypoglycaemia. Both papers suggested that the negative effect of diabetes on mean attainment was greater for earlier diagnosis, though these effects were only found to be significant in Persson. This hints that the mechanisms driving the differences are not equal for all children with diabetes. Persson also found that the differences in attainment between those with and without diabetes are greater for low ability students.

**Limitations**

**Limitations of included studies**

With only two papers based on overlapping datasets, it is difficult to generalise to other populations, particularly when the findings of the included studies relate to a single country with a particularly high prevalence rate of T1DM and excellent diabetes-related outcomes(2).

The included studies controlled for demographics and socio-economic characteristics, but there are other unobserved covariates which should be considered. Other studies considered living arrangements, country of



For peer review only - http://bmjopen.bmj.com/site/about/guidelines.xhtml

birth of the child, main language spoken at home, ability to speak English, attendance in preschool, and parents' occupation.(31) While the included studies were able to adjust for age of diagnosis, they did not have data for other clinical aspects of diabetes, particularly the quality of diabetes management as measured by HbA1c. Cooper et al.(7) were able to show that a higher HbA1c (mean HbA1c during the two years before testing) was associated with poorer attainment, while history of severe hypoglycaemia or history of diabetic ketoacidosis showed no effect on educational attainment.

Finally, neither of the included studies included the secondary outcome, school attendance. In an excluded study, Cooper et al.(7) showed significantly lower school attendance in children with type 1 diabetes in all age groups ($p< 0.001$); from 2.6% lower attendance in year three up to 3.4% lower attendance in year five. This study and that of Crump et al.(32) also used attendance as a covariate in models of attainment.

**Limitations of review**

The primary limitation of the review was that the thresholds for inclusion were very narrow, meaning that only two studies met the inclusion criteria. The small number of studies (with overlapping individuals) meeting the inclusion criteria precluded the use of meta-analysis or other evidence synthesis techniques. While the use of such narrow inclusion criteria was commensurate with the research aim, as well as being consistent with the protocol for the review,(22) future systematic reviewers may consider the way in which the following criteria limited the number of studies included.

The first limiting restriction was including only studies modelling attainment from 'high stakes testing at the end of compulsory schooling'. Although this is justified through the impact of these tests on adult life chances, this criteria excluded studies which have otherwise suitable study designs for exploring the association between diabetes and educational outcomes, such as Cooper et al.(7) This restriction also excluded studies which lack national standardised testing or administrative data records, either because they considered cohorts prior to such testing regimes(33) or were studies from countries without such testing regimes.(34, 35)

The second limiting restriction was excluding publications before 2004 in view of changes in the treatment of T1DM. The 2015 National Institute for Health and Care Excellence (NICE) guidelines(36) state that since 2004 there have been major changes in routine management of type 1 diabetes. Whilst this is pertinent to the UK and most Western countries, this date was perhaps less appropriate for developing counties. In addition, studies published since 2004 were based on cases from earlier years before those changes, for example, the cohorts for the included studies in this review were born 1972-85. This may impact the current applicability of conclusions drawn from these studies, as children with T1DM are likely to now have very different treatment regimens. Both included studies also highlight the change in the school marking system in Sweden in 1998 from a five-level numerical scale to a four-level alphabetical system. Changes to other general examination conditions over this time should also be considered, both in terms of national standardised testing and the use of administrative data records. This shows that more contemporaneous studies are required to assess the impact of advances in treatment alongside current examination conditions and data records. For example, Cooper et al.(7) includes data for children born up to 2003, and so can report insulin pump therapy being associated with better school performance than twice daily injections.

The third limiting restriction was requiring representative control cases which allow estimates that are generalisable to wider populations, for example excluding studies recruiting from a single clinic. As for the restriction on the outcome measure, this excludes countries without registries or linked data. This may lead to



For peer review only - http://bmjopen.bmj.com/site/about/guidelines.xhtml

For peer review only

biases when the countries with high quality linked data, such as the Nordic countries, are also those with high diabetes prevalence.

One related consideration is the use of matching or population controls. In the two included studies these methods are used without justification of the choice or the matching specifications such as the number of controls per case. Matching is traditionally done to make the treatment and control groups more similar. However, these papers do not provide any evidence or reason why those children with type 1 diabetes would be substantively different in terms of other factors which may influence educational attainment. One motivation for the matching approach is to give the appearance of having well-matched healthy controls (regardless of the sparsity of variables used in the process). However, other diagnosed (or undiagnosed) comorbidities within this cohort may affect educational attainment and should be taken into consideration. For example Meo et al.(35) exclude cases with a range of health conditions (reported tobacco use, gross anaemia, tuberculosis, rheumatic fever, rheumatoid arthritis, vision problems, hearing problems or behavioural problems, use of any medication or hospital admissions other than for diabetes mellitus) to reduce the effect of other co-morbidities within the cohort. An alternative to matching with healthy controls is to compare attainment for children with T1DM with children with other health conditions, in order to evaluate the mechanisms through which diabetes affects attainment. For example, asthma may affect sleep and concentration, but have less of an impact on attendance, while epilepsy may share some elements of the social stigma, but have a lower effect on functioning at school.

## CONCLUSION

The studies included in this systematic review show a weak negative association between diabetes and overall grade attainment. In specific subjects, difference in attainment between children with and without T1DM was found to be greatest in Sports/Athletics and smallest in Maths. The negative effect of T1DM was found to be greatest in the lowest quantile of attainment, and findings also suggested lower grade attainment was associated with earlier age at diagnosis. The trend of increasing prevalence of T1DM makes the need for more robust evidence more pressing. More studies from a broader range of contexts are required, including studies utilising alternative methods to provide evidence from countries where large-scale data linkage is not a viable approach. In addition, studies using more contemporaneous data, acknowledging the substantial advances in management of T1DM in the last decade, and ideally linking clinical data such as HbA1c with educational outcomes, are required to aid future recommendations relating to support at school for children with T1DM, especially in the setting of high stakes testing at the end of compulsory schooling.



For peer review only - http://bmjopen.bmj.com/site/about/guidelines.xhtml

## FIGURE 1:

PRISMA 2009 Flow Diagram

## FUNDING

This research was supported in part by the MRC grant MR/N015428/1.

## DUALITY OF INTEREST

All authors have completed the ICMJE uniform disclosure form at www.icmje.org/coi_disclosure.pdf and declare: RF has received a grant from the Medical Research Council MR/N015428/1 for his work as principal investigator of the project 'Investigating the inter-relationship between diabetes and children's educational achievement'. All authors have no conflict of interest to report; no financial relationships with any organisations that might have an interest in the submitted work in the previous three years; no other relationships or activities that could appear to have influenced the submitted work.

## CONTRIBUTION STATEMENT

RF is the review guarantor. The concept of the review was proposed by RF and JG; the manuscript was drafted by NO and RF, and edited by all authors. MH, NO and RF designed the search strategy with advice from MM. NO, MM and RF contributed to the development of the study eligibility criteria and data extraction criteria. JG and CD provided expertise on type 1 diabetes. MM provided expertise on systematic review methodology. NO and RF screened the titles and abstracts of records retrieved from the searches using the predetermined inclusion criteria, with any doubt or disagreement discussed with JG. NO and RF independently completed data extraction and assessed the quality and risk of bias of the included studies using the Newcastle-Ottawa Scale (NOS), consulting JG if any disagreements. DK provided expertise on data extraction & analysis. All authors read, edited and approved the final manuscript.

## DATA AVAILABILITY STATEMENT

All data relevant to the study are included in the article or uploaded as supplementary information.



For peer review only - http://bmjopen.bmj.com/site/about/guidelines.xhtml

# REFERENCES


1. Holt R, Cockram C, Flyvbjerg A, Goldstein B. Textbook of Diabetes, 5th edition. West Sussex, UK: John Wiley and Sons Ltd; 2017.
2. International Diabetes Federation. IDF Diabetes Atlas, 8th edition. Brussels, Belgium: International Diabetes Federation; 2017. Available from: http://www.diabetesatlas.org. (Accessed 30th August 2018)
3. Persson S, Dahlquist G, Gerdtham UG, Steen Carlsson K. Impact of childhood-onset type 1 diabetes on schooling: a population-based register study. *Diabetologia* 2013;56(6):1254-62.
4. Streisand R, Monaghan M. Young Children with Type 1 Diabetes: Challenges, Research, and Future Directions. *Current diabetes reports* 2014;14(9):520.
5. Graveling AJ, Deary IJ, Frier BM. Acute Hypoglycemia Impairs Executive Cognitive Function in Adults With and Without Type 1 Diabetes. *Diabetes Care* 2013;36(10):3240-6.
6. Cameron FJ, Scratch SE, Nadebaum C, Northam EA, Koves I, Jennings J et al. Neurological Consequences of Diabetic Ketoacidosis at Initial Presentation of Type 1 Diabetes in a Prospective Cohort Study of Children. *Diabetes Care* 2014;37(6):1554-62.
7. Cooper MN, McNamara KA, de Klerk NH, Davis EA, Jones TW. School performance in children with type 1 diabetes: A contemporary population-based study. *Pediatric Diabetes* 2016;17(2):101-11.
8. Chowdry H, Crawford C, et al. Widening participation in higher education: analysis using linked administrative data. *Journal of the Royal Statistical Society Series A Statistics in Society* 2013;176(2):431-57.
9. Marchant G. What is at Stake with High Stakes Testing? A Discussion of Issues and Research. *The Ohio Journal of Science* 2004;104(2):2-7.
10. Amillategui B, Calle JR, Alvarez MA, Cardiel MA, Barrio R. Identifying the special needs of children with Type 1 diabetes in the school setting. An overview of parents' perceptions. *Diabetic medicine : a journal of the British Diabetic Association* 2007;24(10):1073-9.
11. Desrocher M, Rovet J. Neurocognitive correlates of type 1 diabetes mellitus in childhood. *Child Neuropsychol* 2004;10:36-52.
12. Gaudieri P et al. Cognitive function in children with type 1 diabetes: a meta-analysis. *Diabetes Care* 2008;31:1892-7.
13. Naguib J et al. Neuro-cognitive performance in children with type 1 diabetes- a meta-analysis. *J Pediatr Psychol* 2009;34:271-82.
14. He J et al. Glycemic extremes are related to cognitive dysfunction in children with type 1 diabetes: A meta-analysis. *J Diabetes Investig* 2018;9(6):1342–53.
15. Diabetes UK. Diabetes in schools - legal information 2017. Available from: https://www.diabetes.org.uk/guide-to-diabetes/your-child-and-diabetes/schools/diabetes-in-schools-legal-information. (Accessed 20th August 2018)
16. Her Majesty's Stationery Office (HMSO). Equality Act 2010 (England, Scotland, Wales) 2010. Available from: http://www.legislation.gov.uk/ukpga/2010/15/pdfs/ukpga_20100015_en.pdf (Accessed 20th August 2018)
17. Her Majesty's Stationery Office (HMSO). Children and Families Act 2014 (England) 2014. Available from: http://www.legislation.gov.uk/ukpga/2014/6/pdfs/ukpga_20140006_en.pdf (Accessed 20th August 2018)
18. Her Majesty's Stationery Office (HMSO). Additional Learning Needs and Education Tribunal Act (Wales) 2018. Available from: http://www.legislation.gov.uk/anaw/2018/2/pdfs/anaw_20180002_en.pdf (Accessed 20th August 2018)
19. Her Majesty's Stationery Office (HMSO). Education (Additional Support For Learning) Act (Scotland) 2004. Available from: https://www.legislation.gov.uk/asp/2004/4/pdfs/asp_20040004_en.pdf (Accessed 20th August 2018)
20. Department of Education and Department of Health Social Services and Public Safety. Supporting Pupils With Medication Needs (Northern Ireland) 2008. Available from: *https://www.education-ni.gov.uk/sites/default/files/publications/de/supporting-pupils-with-medical-needs.pdf* (Accessed 20th August 2018)




For peer review only - http://bmjopen.bmj.com/site/about/guidelines.xhtml

21. Oakley N, Kneale D, Mann M, Hilliar M, Tan J, Dayan C et al. The Association between Type 1 Diabetes Mellitus and Educational Attainment in Childhood: A Systematic Review. PROSPERO International Prospective Register of Systematic Reviews 2017 CRD42017084078. Available from: http://www.crd.york.ac.uk/PROSPERO/display_record.php?ID=CRD42017084078 (Accessed 20th August 2018)
22. Oakley N, Kneale D, Mann M et al. Association between type 1 diabetes mellitus and educational attainment in childhood: a systematic review protocol. *BMJ Open* 2018;8:e021893.
23. Higgins J, Green S. Cochrane Handbook for Systematic Reviews of Intervention. Version 5.1.0 [updated March 2011]. The Cochrane Collaboration. Available from: http://handbook-5-1.cochrane.org/ (Accessed 20th August 2018)
24. Moher D, Liberati A, Tetzlaff J, Altman D, The PRISMA Group. Preferred Reporting Items for Systematic Reviews and Meta-Analyses: The PRISMA Statement. *PLoS Medicine* 2009;6(7):e1000097.
25. (NIH) U.S. National Library of Medicine. ClinicalTrials.gov 2018. Available from: https://clinicaltrials.gov/ (Accessed 20th August 2018)
26. BioMed Central Ltd. International Standard Randomised Controlled Trial Number (ISRCTN) Registry 2018. Available from: http://www.isrctn.com/ (Accessed 20th August 2018)
27. World Health Organisation. International Clinical Trials Registry Platform (ICTRP). Available from: http://www.who.int/ictrp/en/ (Accessed 20th August 2018)
28. Thomas J, Brunton J, Graziosi S. EPPI-Reviewer 4: software for research synthesis. EPPI-Centre Software. London: Social Science Research Unit, UCL Institute of Education; 2010.
29. Schünemann H, Brożek J, Guyatt G, Oxman A (eds). GRADE handbook for grading quality of evidence and strength of recommendations. Updated October 2013: The GRADE Working Group. Available from: www.guidelinedevelopment.org/handbook. (Accessed 20th August 2018)
30. Dahlquist G, Kallen B. School performance in children with type 1 diabetes-a population-based register study. *Diabetologia* 2007;50(5):957-64.
31. Nasuuna E et al. Examining the relationship between childhood health conditions and health service utilisation at school entry and subsequent academic performance in a large cohort of Australian children. *Journal of Paediatrics and Child Health* 2016;52:750-8.
32. Crump C, Rivera D, London R, Landau M, Erlendson B, Rodriguez E. Chronic health conditions and school performance among children and youth. *Annals of Epidemiology* 2013;23(4):179-84.
33. von Stumm S et al. Childhood behavior problems and health at midlife: 35-year follow-up of a Scottish birth cohort. *Journal of child psychology and psychiatry and allied disciplines* 2011;52(9):992-1001.
34. Elrayah H et al. Economic burden on families of childhood type 1 diabetes in urban Sudan. *Diabetes Research and Clinical Practice* 2005;70(2):159-65.
35. Meo SA, Alkahlan MA, Al-Mubarak MA, Al-Obayli MS, Melaibary BA, Bin Dous AN et al. Impact of type 1 diabetes mellitus on academic performance. *Journal of International Medical Research* 2013;41(3):855-8.
36. National Institute of Clinical Excellence. NICE Guideline [NG18] Diabetes (Type 1 and Type 2) in children and young people: diagnosis and management; 2015. Available from: https://www.nice.org.uk/guidance/ng18. (Accessed 20th August 2018)



For peer review only - http://bmjopen.bmj.com/site/about/guidelines.xhtml

# Figure 1

## PRISMA 2009 Flow Diagram

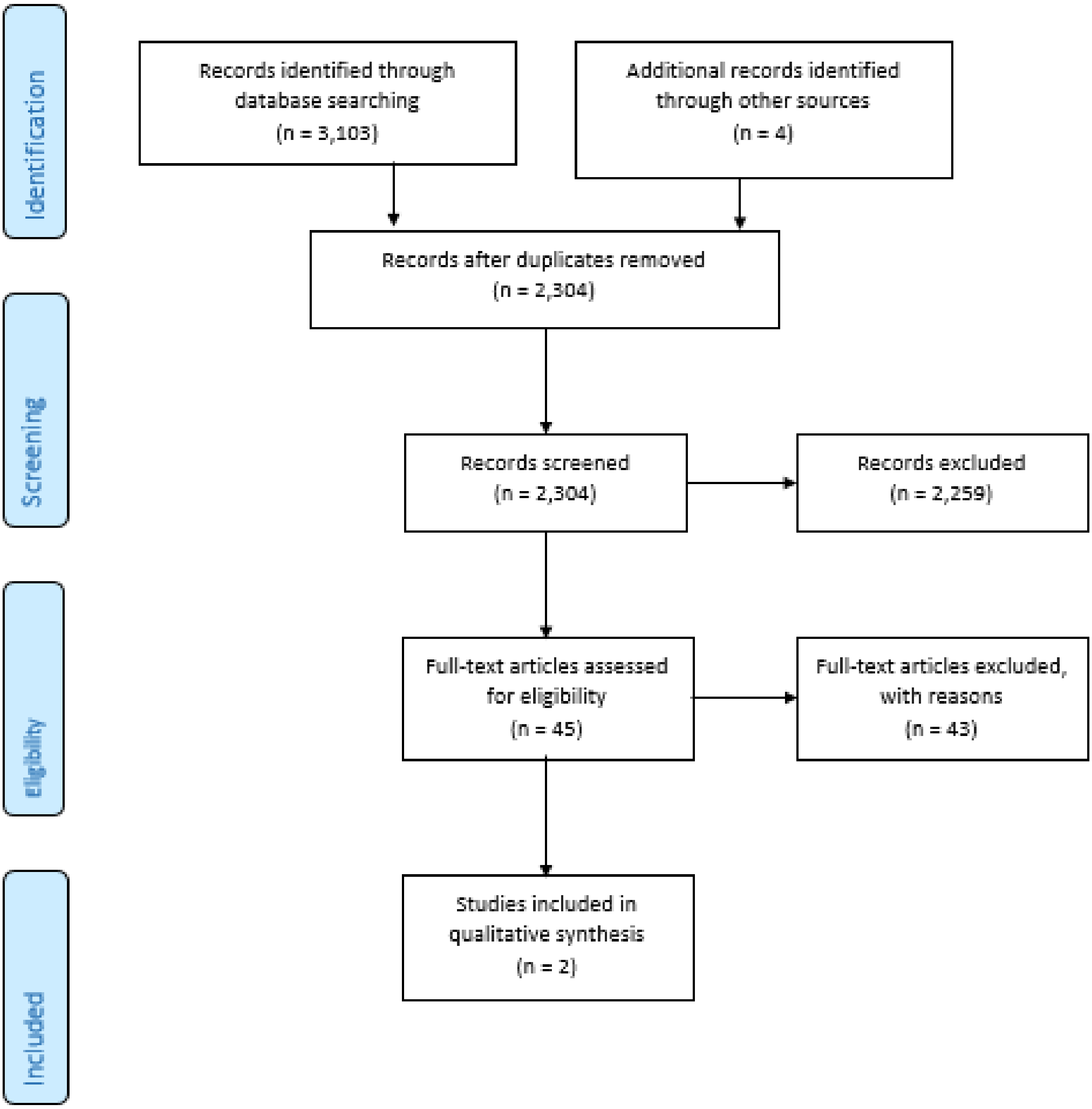


*From:* Moher D, Liberati A, Tetzlaff J, Altman DG, The PRISMA Group (2009). *P*referred *R*eporting *I*tems for *S*ystematic Reviews and *M*eta-*A*nalyses: The PRISMA Statement. PLoS Med 6(7): e1000097. doi:10.1371/journal.pmed1000097

**Appendix 1: Search Strategies**

Search Strategy for Ovid MEDLINE (R) (1946 to present), Ovid MEDLINE(R) Epub Ahead of Print, In-Process & Other Non-Indexed Citations, Ovid EMBASE (1947 to present).

| | Searches |
|---|---|
| 1 | exp Child/ |
| 2 | exp Pediatrics/ |
| 3 | exp Adolescent/ |
| 4 | teen*.ti,ab. |
| 5 | child*.ti,ab. |
| 6 | adolescen*.ti,ab. |
| 7 | p?ediatric*.ti,ab. |
| 8 | juvenile*.ti,ab. |
| 9 | youth*.ti,ab. |
| 10 | (young adj3 (person* or people)).ti,ab. |
| 11 | minors.ti,ab. |
| 12 | or/1-11 |
| 13 | exp Diabetes Mellitus, Type 1/ |
| 14 | (type 1 diabetes or T1D or T1DM or diabet*).ti,ab. |
| 15 | (Insulin adj3 dependent).ti,ab. |
| 16 | 13 or 14 or 15 |
| 17 | (academic* adj3 (attain* or grade* or performance* or success* or status* or outcome* or result* or mark* or achiev* or score* or progress*)).ti,ab. |
| 18 | (educat* adj3 (attain* or grade* or performance* or success* or outcome* or result* or status* or mark* or achiev* or score* or progress*)).ti,ab. |
| 19 | (school* adj3 (attain* or grade* or performance* or success* or status* or outcome* or result* or mark* or achiev* or score* or progress*)).ti,ab. |
| 20 | exp Educational Status/ |
| 21 | or/17-20 |
| 22 | 12 and 16 and 21 |
| 23 | limit 22 to yr="2004 -Current" |

For peer review only

Search Strategy for Thomson Reuters Web of Science

| | Searches |
|---|---|
| #4 | #3 AND #2 AND #1 (Timespan=2004-present) |
| #3 | TS=(type 1 diabetes or t1d or t1dm or diabet* or insulin near/3 dependent) |
| #2 | TS=(((academic* or educat* or school*) near/3 (attain* or grade* or performance* or success* or status* or outcome* or result* or mark* or achiev* or score* or progress*))) |
| #1 | TS=(child* or p?ediatric or juvenile* or adolescen* or teen* or minors or juvenile or youth* or (young near/3 (people or person*))) |

Search Strategy for EBSCO British Education Index (BEI), EBSCO Education Resources Information Center (ERIC) & EBSCO Cumulative Index to Nursing and Allied Health Literature (CINAHL)

| | Searches |
|---|---|
| S10 | S3 AND S6 AND S9 (Limiter=2004-present) |
| S9 | S7 OR S8 |
| S8 | TI ((academic* or educat* or school*) N3 (attain* or grade* or performance* or success* or status* or outcome* or result* or mark* or achiev* or score* or progress*)) |
| S7 | AB ((academic* or educat* or school*) N3 (attain* or grade* or performance* or success* or status* or outcome* or result* or mark* or achiev* or score* or progress*)) |
| S6 | S4 OR S5 |
| S5 | AB child* or p?ediatric or adolescen* or teen* or minors or juvenile* or youth* or (young N3 (people or person*) |
| S4 | TI child* or p?ediatric or adolescen* or teen* or minors or juvenile* or youth* or (young N3 (people or person*) |
| S3 | S1 OR S2 |
| S2 | AB(diabet* or type 1 diabetes or T1D or T1DM) |
| S1 | TI(diabet* or type 1 diabetes or T1D or T1DM) |

For peer review only

**Appendix 2:**

| **Journal title** |
|---|
| New England Journal of Medicine |
| Lancet |
| JAMA |
| Lancet Internal Medicine |
| BMJ |
| Diabetes Care |
| PLOS Medicine |
| Diabetologia |
| Pediatric Diabetes |
| Endocrine Reviews |
| Nature Reviews Endocrinology |
| Trends in Endocrinology & Metabolism |
| Diabetes |
| Journal of Clinical Endocrinology & Metabolism |
| Diabetes, Obesity & Metabolism |
| American Journal of Physiology – Endocrinology and Metabolism |
| Reviews in Endocrine & Metabolic Disorders |
| Journal of Endocrinology |
| European Journal of Endocrinology |
| Diabetes/ Metabolism Research and Reviews |
| Best Practice and Research in Clinical Endocrinology and Metabolism |
| Diabetes and Metabolism Journal |

**Table 1: List of key journals for which electronic table of contents were searched for additional relevant articles.**

For peer review only

**Appendix 3:**

| Excluded Studies |
|---|
| Abusrewil S. (2013) Epidemiology of childhood diabetes in western part of Libya (1989-2013). Pediatric Diabetes 18:117. |
| Almqvist C et al. (2016) Sibship and risk of asthma in a total population: A disease comparative approach. Journal of Allergy and Clinical Immunology 138(4):1219-1222e3. |
| Bass L (2011) Social Focus on Health and Children's Well-being. Sociological Inquiry 81: 495-498 |
| Bezerra A et al. (2012) School attainment in childhood is an independent risk factor of dementia in late life: results from a Brazilian sample. International Psychogeriatrics 24(1): 55-61. |
| Bjerkedal T et al. (2006) A follow up of persons who received basic and/or supplemental benefits in childhood. Tidsskr Nor Legeforen 126: 436-439 |
| Bortes C et al. (2018) Health problems during childhood and school achievement: Exploring associations between hospitalization exposures, gender, timing, and compulsory school grades. PLoS ONE 13(12):e0208116. |
| Catalano D et al. (2004) Journal of Allergy and Clinical Immunology. 155(5): 175-8. |
| Champaloux S, Young D (2015) Childhood Chronic Health Conditions and Educational Attainment: A Social Ecological Approach. Journal of Adolescent Health 56: 98-105 |
| Chaudhry T, Klonoff D (2013) SMBG out of control: the need for educating patients about control solution. Diabetes Educator 39: 689-695 |
| Compas B, et al. (2017) Neurocognitive Deficits in Children With Chronic Health Conditions. American Psychologist 72: 326-338 |
| Cooper MN, McNamara KA, de Klerk NH, Davis EA, Jones TW (2016) School performance in children with type 1 diabetes: A contemporary population-based study. Pediatric Diabetes 17: 101-111. |
| Crump C, Rivera D, London R, Landau M, Erlendson B, Rodriguez E (2013) Chronic health conditions and school performance among children and youth. Annals of Epidemiology 23: 179-184. |
| Elrayah H, et al. (2005) Economic burden on families of childhood type 1 diabetes in urban Sudan. Diabetes Research and Clinical Practice 70: 159-165 |
| Engelke M et al. (2008) School nurse case management for children with chronic illness: health, academic, and quality of life outcomes. Journal of School Nursing 24(4):205-214 |
| Erkolahti R, Ilonen T (2005) Academic Achievement and the Self-Image of Adolescents with Diabetes Mellitus Type-1 And Rheumatoid Arthritis. Journal of Youth and Adolescence 34: 199. |
| Fraser A et al. (2012) Associations of existing diabetes, gestational diabetes, and glycosuria with offspring IQ and educational attainment: The avon longitudinal study of parents and children. Experimental Diabetes Research 963735. |
| Gonzalez A, et al. (2012) Childhood and family influences on depression, chronic physical conditions, and their comorbidity: Findings from the Ontario Child Health Study. Journal of Psychiatric Research 46: 1475-1482 |
| Hassan M et al. (2017) Assessment of health-related quality of life in Egyptian adolescents with type 1 diabetes: DEMPU survey. Journal of Pediatric Endocrinology and Metabolism 30(3): 277-283. |
| Jameson P et al. (2006) Diabetes, cognitive function, and school performance. School Nurse News 23(3):34-36. |
| Jesic M et al. (2013) School problems in children and adolescents with type 1 diabetes. Pediatric Diabetes 18:102-103. |
| Lansing A et al. (2018) Academic achievement and metabolic control in adolescents with type 1 diabetes, Children's Health Care 47(1):16-33 |
| Liaqat A at al. (2017) Epidemology of type-I diabetes and its economic burden on society. Pakistan Journal of Medical and Health Sciences 11(2):714-717. |
| Lynch P et al. (2004) Predicting academic achievement in youths with type 1 diabetes: A biopsychosocial model. Diabetes 53:A436. |
| Maslow G et al. (2011) Growing up with a chronic illness: social success, educational/vocational distress. Journal of Adolescent Health 49(2):206-12. |
| Maslow G et al. (2012) Protective connections and educational attainment among young adults with childhood-onset chronic illness. 82(8):364-70. |

For peer review only

| |
|---|
| Meo SA, Alkahlan MA, Al-Mubarak MA, et al. (2013) Impact of type 1 diabetes mellitus on academic performance. Journal of International Medical Research 41: 855-858 |
| Merrick H et al. (2015) Characteristics of young people with long term conditions close to transfer to adult health services. BMC health services research 15:435. |
| Milovanovic I et al. (2012) Entred-Ado study: Health, education and risk behaviours of adolescents with diabetes. Diabetologia 1:S140. |
| Mitosi N et al. (2013) Family role in treatment adherence of type 1 diabetes: Research data from Greek population. Pediatric Diabetes 18:110. |
| Nasuuna E, Santoro G, Kremer P, de Silva AM (2016) Examining the relationship between childhood health conditions and health service utilisation at school entry and subsequent academic performance in a large cohort of Australian children. Journal of Paediatrics and Child Health 52: 750-758 |
| Neves C et al. (2013) Obesity, diet and physical activity: The reality of our teenagers. Atencion Primaria 2:84. |
| Nielson H et al. (2016) Type 1 diabetes, quality of life, occupational status and education level - A comparative population-based study. Diabetes Research & Clinical Practice 121:62-68 |
| Ovesen L et al. (2015) Education level, occupational status and quality of life in adults with type 1 diabetes and in the general population: A comparative study. Diabetologia 1:S412. |
| Roman R et al. (2016) School performance in children and adolescents with type 1 diabetes. Hormone Research in Paediatrics 86:51. |
| Roman R et al. (2017) Metabolic control and school performance in children with type 1 diabetes. Revista Chilena de Pediatria 88(5):586-594. |
| Ryan C. (2012) Does severe hypoglycaemia disrupt academic achievement in children with early onset diabetes? Developmental Medicine and Child Neurology 54(5):393-394 |
| Schiel R et al. (2016) Plasma copeptin in children and adolescents with type 1 diabetes mellitus in comparison to healthy controls. Diabetes Research and Clinical Practice 118:156-161. |
| Steen Carlsson K et al. (2015) Type 1 diabetes with early onset and educational field at upper secondary and university level: Is own experience an asset for a health care carrier? Diabetologia 1:S411. |
| Tahirovic H et al. (2013) Impact of socioeconomic status on health-related quality of life in children with type 1 diabetes mellitus in Bosnia and Herzegovina. Minerva Pediatrica 65(2):207-212. |
| Taras H et al. (2005) Chronic health conditions and student performance at school. Journal of School Health 75(7):255-266. |
| von Stumm S et al. (2011) Childhood behavior problems and health at midlife: 35-year follow-up of a Scottish birth cohort. Journal of child psychology and psychiatry and allied disciplines 52(9):992-1001. |
| Wennick A et al. (2011) Attained education and self-assessed health later in life when diagnosed with diabetes in childhood: a population-based study. Pediatric Diabetes 12(7):619-26. |
| Winnick J et al. (2017) Metabolic Control and Academic Achievement Over Time Among Adolescents With Type 1 Diabetes. School Psychology Quarterly 32(1):105-117. |

**Table 1: List of excluded studies following full text review.**

For peer review only

**Appendix 4:**

| | Newcastle Ottawa Scale | | | | Overall Risk of Bias |
|---|---|---|---|---|---|
| **Study** | **Selection** | **Comparability** | **Outcome** | **Overall** | |
| **Dahlquist & Kallen (D2007)** | 4/4 | 2/2 | 2/3 | **8/9** | **Low** |
| **Persson et al. (D2013)** | 4/4 | 2/2 | 3/3 | **9/9** | **Low** |

**Table 1: Newcastle Ottawa Scale ratings for included studies.**

For peer review only

# PRISMA 2009 Checklist

| Section/topic | # | Checklist item | Reported on page # |
|---|---|---|---|
| **TITLE** | | | |
| Title | 1 | Identify the report as a systematic review, meta-analysis, or both. | 1 |
| **ABSTRACT** | | | |
| Structured summary | 2 | Provide a structured summary including, as applicable: background; objectives; data sources; study eligibility criteria, participants, and interventions; study appraisal and synthesis methods; results; limitations; conclusions and implications of key findings; systematic review registration number. | 2 |
| **INTRODUCTION** | | | |
| Rationale | 3 | Describe the rationale for the review in the context of what is already known. | 3, 4 |
| Objectives | 4 | Provide an explicit statement of questions being addressed with reference to participants, interventions, comparisons, outcomes, and study design (PICOS). | 5 |
| **METHODS** | | | |
| Protocol and registration | 5 | Indicate if a review protocol exists, if and where it can be accessed (e.g., Web address), and, if available, provide registration information including registration number. | 2, 5 |
| Eligibility criteria | 6 | Specify study characteristics (e.g., PICOS, length of follow-up) and report characteristics (e.g., years considered, language, publication status) used as criteria for eligibility, giving rationale. | 5, 6, Protocol |
| Information sources | 7 | Describe all information sources (e.g., databases with dates of coverage, contact with study authors to identify additional studies) in the search and date last searched. | 5, 6, Appendix 1+2 |
| Search | 8 | Present full electronic search strategy for at least one database, including any limits used, such that it could be repeated. | 5, Appendix 1 |
| Study selection | 9 | State the process for selecting studies (i.e., screening, eligibility, included in systematic review, and, if applicable, included in the meta-analysis). | 5, Protocol |
| Data collection process | 10 | Describe method of data extraction from reports (e.g., piloted forms, independently, in duplicate) and any processes for obtaining and confirming data from investigators. | 5, 6, Protocol |
| Data items | 11 | List and define all variables for which data were sought (e.g., PICOS, funding sources) and any assumptions and simplifications made. | Protocol |
| Risk of bias in individual studies | 12 | Describe methods used for assessing risk of bias of individual studies (including specification of whether this was done at the study or outcome level), and how this information is to be used in any data synthesis. | 5, 6 |
| Summary measures | 13 | State the principal summary measures (e.g., risk ratio, difference in means). | 6 |
| Synthesis of results | 14 | Describe the methods of handling data and combining results of studies, if done, including measures of consistency (e.g., $I^2$) for each meta-analysis. | 6 |

For peer review only

For peer review only - http://bmjopen.bmj.com/site/about/guidelines.xhtml

# PRISMA 2009 Checklist

| Section/topic | # | Checklist item | Reported on page # |
|---|---|---|---|
| Risk of bias across studies | 15 | Specify any assessment of risk of bias that may affect the cumulative evidence (e.g., publication bias, selective reporting within studies). | 5, 6 |
| Additional analyses | 16 | Describe methods of additional analyses (e.g., sensitivity or subgroup analyses, meta-regression), if done, indicating which were pre-specified. | 6 |
| **RESULTS** | | | |
| Study selection | 17 | Give numbers of studies screened, assessed for eligibility, and included in the review, with reasons for exclusions at each stage, ideally with a flow diagram. | 6, 7, Figure 1, Table 1, Appendix 3 |
| Study characteristics | 18 | For each study, present characteristics for which data were extracted (e.g., study size, PICOS, follow-up period) and provide the citations. | 7, 8, 14, 15, Table 2. |
| Risk of bias within studies | 19 | Present data on risk of bias of each study and, if available, any outcome level assessment (see item 12). | 8-10, Table 4, Appendix 4. |
| Results of individual studies | 20 | For all outcomes considered (benefits or harms), present, for each study: (a) simple summary data for each intervention group (b) effect estimates and confidence intervals, ideally with a forest plot. | 6-10, Tables 3+4. |
| Synthesis of results | 21 | Present results of each meta-analysis done, including confidence intervals and measures of consistency. | N/A |
| Risk of bias across studies | 22 | Present results of any assessment of risk of bias across studies (see Item 15). | 6-10, Table 4, Appendix 4 |
| Additional analysis | 23 | Give results of additional analyses, if done (e.g., sensitivity or subgroup analyses, meta-regression [see Item 16]). | 6-10, Table 3 |
| **DISCUSSION** | | | |
| Summary of evidence | 24 | Summarize the main findings including the strength of evidence for each main outcome; consider their relevance to key groups (e.g., healthcare providers, users, and policy makers). | 8-10, Tables 3+4 |
| Limitations | 25 | Discuss limitations at study and outcome level (e.g., risk of bias), and at review-level (e.g., incomplete retrieval of identified research, reporting bias). | 10-12 |
| Conclusions | 26 | Provide a general interpretation of the results in the context of other evidence, and implications for future research. | 10-12 |
| **FUNDING** | | | |
| Funding | 27 | Describe sources of funding for the systematic review and other support (e.g., supply of data); role of funders for the systematic review. | 13 |

*From:* Moher D, Liberati A, Tetzlaff J, Altman DG, The PRISMA Group (2009). Preferred Reporting Items for Systematic Reviews and Meta-Analyses: The PRISMA Statement. PLoS Med 6(7): e1000097. doi:10.1371/journal.pmed1000097

For more information, visit: **www.prisma-statement.org**

For peer review only - http://bmjopen.bmj.com/site/about/guidelines.xhtml



For peer review only
